# Network Analysis Reveals Ecological Links between N-Fixing Bacteria and Wood-Decaying Fungi

**DOI:** 10.1371/journal.pone.0088141

**Published:** 2014-02-05

**Authors:** Björn Hoppe, Tiemo Kahl, Peter Karasch, Tesfaye Wubet, Jürgen Bauhus, François Buscot, Dirk Krüger

**Affiliations:** 1 Department of Soil Ecology, UFZ - Helmholtz Centre for Environmental Research, Halle (Saale), Germany; 2 Institute of Silviculture, Faculty of Environment and Natural Resources, University of Freiburg, Freiburg i. Brsg., Germany; 3 Pilzteam Bayern, Hohenau, Germany; 4 Institute of Biology I, University of Leipzig, Leipzig, Germany; 5 The German Centre for Integrative Biodiversity Research (iDiv), University Leipzig, Leipzig, Germany; INRA Clermont-Ferrand Research Center, France

## Abstract

Nitrogen availability in dead wood is highly restricted and associations with N-fixing bacteria are thought to enable wood-decaying fungi to meet their nitrogen requirements for vegetative and generative growth. We assessed the diversity of *nifH* (dinitrogenase reductase) genes in dead wood of the common temperate tree species *Fagus sylvatica* and *Picea abies* from differently managed forest plots in Germany using molecular tools. By incorporating these genes into a large compilation of published *nifH* sequences and subsequent phylogenetic analyses of deduced proteins we verified the presence of diverse pools corresponding to functional *nifH*, almost all of which are new to science. The distribution of *nifH* genes strongly correlated with tree species and decay class, but not with forest management, while higher fungal fructification was correlated with decreasing nitrogen content of the dead wood and positively correlated with *nifH* diversity, especially during the intermediate stage of wood decay. Network analyses based on non-random species co-occurrence patterns revealed interactions among fungi and N-fixing bacteria in the dead wood and strongly indicate the occurrence of at least commensal relationships between these taxa.

## Introduction

Dead wood is an important structural component of forest ecosystems. It influences numerous ecosystem functions [1], [2], including carbon (C) sequestration [3]–[6], nutrient cycling [7], and provision of habitats for wood-dwelling organisms [8], [9]. Although many investigations have focused on the diversity of fungi, particularly Agaricomycotina (basidiomycetes) and some Pezizomycotina (ascomycetes), in terms of assemblages [10]–[12] or their role in wood decomposition [13], the participation of bacteria in the processes involved is largely unexplored. It is known that substrate qualities, such as nutrient and water contents, strongly influence wood colonization by microbes [14]. The amount of nitrogen (N) available in wood is highly restricted [1], with carbon to nitrogen ratios generally ranging from ca. 350–800:1 [15], but wood-decaying fungi can completely mineralize and metabolize most wood residues such as cell wall lignocellulose complexes [16] and are capable of mobilizing enough N to produce not only their vegetative hyphae but also sporocarps and millions of spores. Cowling and Merrill [17] hypothesized that associations with N-fixing bacteria may enable wood-inhabiting fungi to meet their substantial N requirements. Nitrogen fixation, the energetically expensive reduction of atmospheric dinitrogen to two molecules of ammonia, is enabled by adenosine triphosphate (ATP) generated from the decomposition of cellulose [18]. This connects bacterial colonization of dead wood with fungal processes of wood decay. The presence and activity of N-fixing bacteria in both living and dead wood have been assessed by acetylene reduction assays in various studies [7], [15], [19]–[22]. However, with advances of molecular techniques in the 1980s, Zehr and McReynolds [23] were the first to establish oligonucleotide primers to amplify the *nifH* gene complex that encodes dinitrogenase reductase. *NifH* is still the standard target in studies on N-fixing prokaryotes in various natural environments [24]. Many investigations have been conducted on the molecular ecology of diazotrophic (N-fixing) communities in nitrogen-limited substrates, such as forest soils, salt marshes and oligotrophic marine sediments [25]. Wang et al. [26] recently reported on the distribution of *nifH* genes in four terrestrial climatic zones across the USA, where they surprisingly discovered an 80% overlap on the 95% amino acid identity threshold among their and already known genes. We are, however lacking information on *nifH* gene distribution in dead wood to date. In addition, several authors have investigated the diversity and community structure of bacteria in dead wood as well as functional traits related to white-rot fungi [27], [28], but without focusing on diazotrophic bacteria. The work presented here emanated from the initial idea to survey bacterial community structure on dead wood to gain information whether potential N-fixers are present or not. This prompted us to immediately investigate the presence and distribution of *nifH* genes in dead wood. The objectives of the present study were to: a) explore the diversity of *nifH* sequences in dead wood, b) test the hypothesis that the community composition of N-fixing bacteria correlates with the diversity of fungi fructifying on dead wood, and c) assess the likelihood that these sequences encode functional enzymes by phylogenetic methods.

## Materials and Methods

### Experimental Design

The study plots are located in the UNESCO Biosphere Reserve “Schwäbische Alb” in southern Germany, one of three experimental sites included in the German Biodiversity Exploratories [29], designed to provide a large-scale, long-term open platform for functional biodiversity research along a north-south gradient in Germany. The main objectives of this endeavor are to elucidate the influence of land use and management type on biodiversity and ecosystem functioning. The mean annual temperature at the “Schwäbische Alb” exploratory is 6–7°C and annual precipitation ranges between ca. 700 and 1000 mm. Our survey was conducted on dead wood logs (hereafter “logs”) in very intensively investigated 1 ha plots (VIP), representing the following three forest management types: (i) extensively managed beech forests, where timber harvesting stopped several decades ago, (ii) managed beech forests dominated by *Fagus sylvatica* and (iii) managed spruce forests dominated by *Picea abies*.

### Dead Wood

In April 2009, logs located on the forest floor in the VIPs were randomly selected and their properties (length, diameter, tree species) were characterized. For the present study on N-fixing bacterial genes, subsets of dead wood logs were randomly selected representing each of the two focal tree species (*P. abies* and *F. sylvatica*) in plots representing the three management types. The random subset selection assured that *Fagus* logs were present in *Picea*-dominated plots and *vice versa*, giving 45 logs in total (a sampling scheme is displayed in Fig. S1). In June 2009 wood chips from the logs were sampled using a cordless Makita BDF451 drill (Makita, Anja, Japan) equipped with a 2×42 cm wood auger. The number of cores drilled depended on the volume and length of the logs. A minimum of three cores were drilled up to a log length of 5 m. Each additional 5 m of log length resulted in another drill core. A maximum of 7 cores was sampled in a 25 m long log. To avoid contamination between samples, the wood auger was flamed and wiped with ethanol between each core. The drill was operated slowly and introduced at an angle of ∼45° to a line perpendicular to the log axis. To avoid overheating the sample, the operation was paused periodically. Depending on the log’s diameter at the point of drilling, the auger was either drilled through it or inserted to its maximum length. The wood samples were kept on dry ice and later stored at –80°C upon return to the lab. The total volume of wood chips from each drill core was ground under liquid nitrogen into a fine powder using a Retsch MM400 swing mill (Retsch, Haan, Germany). Wood C and N concentrations were determined through total combustion. For this purpose 10 mg of each wood sample was weighed into a tin capsule and analyzed using a Truspec elemental analyzer (Leco, St. Joseph, MI, USA). The remaining mass after decay (%) of each log was calculated using information on its dimensions (length, diameter and volume loss), density and the density of fresh undecayed wood, according to Kahl et al. [5]. Dead wood logs were assigned to 4 decay classes based on remaining mass (%) data by k-means cluster analysis [30] in R v. 2.11.1. This classification is more reliable than the conventional method of educated guessing by an observer.

### Fungal Sporocarp Inventories

Sporocarps are part of the currently actively growing fungi in the wood substrate and were chosen as study objects rather than conducting a more comprehensive nucleic acid based assessment of fungal diversity that may include inactive fungi. Sampling of sporocarps took place on the preselected subset of dead wood logs at three different occasions in June (contemporary with wood sampling for molecular analyses of *nifH* genes), September and October/November 2009 to cover the full aspect of fructification of particular species according to their phenology across the course of the year. All sporocarps larger then 1 cm were sampled, excluding fully resupinate corticoid fungi (Basidiomycota) and non-stromatic pyrenomycetes and discomycetes of the phylum Ascomycota and morphologically identified to the species level if possible. Dried specimens were deposited at the herbarium LZ (University Leipzig).

### DNA Isolation

Total community DNA from 1 g of each previously homogenized wood sample, which was divided into four 1.5 ml microcentrifuge tubes, was isolated using a modified CTAB-protocol [31]. Briefly, 900 µl of 2x CTAB buffer (2% [wt/vol] hexadecyltrimethylammonium bromide; 100 mM TrisHCl, pH 8.0; 1.4 M NaCl; 20 mM EDTA; 1.5% polyvinyl-pyrrolidone (PVP), 0.2% [vol/vol] beta-mercaptoethanol), was added to the sample. Tubes were incubated at 55°C for one hour. Nucleic acids were separated from proteins and cell debris by adding 500 µl of 24:1 chloroform:isoamyl alcohol and subsequent centrifugation at maximum speed for 10 min followed by another 500 µl chloroform addition and centrifugation at maximum speed for 5 min. DNA was precipitated with 0.08 volumes of 7.5 M ammonium acetate and 0.54 volumes of isopropanol and washed twice with 99% ethanol. Dried DNA pellets were dissolved in 100 µl molecular grade water (all chemicals supplied by Merck, Darmstadt, Germany and Carl Roth, Karlsruhe, Germany).

### PCR, Cloning and Initial Sequence Analysis

All DNA extracts from the wood samples of each log were pooled into a composite extract prior to PCR. The primer pair PolF (5'- TGC GAY CCS AAR GCB GAC TC -3') and PolR (5’- ATS GCC ATC ATY TCR CCG GA -3') [32] was used to amplify a 360 bp fragment of the *nifH* gene. Each composite DNA extract was amplified separately by PCR in triplicate 20 µl reaction mixtures containing 4 µl FIREPol 5x Master Mix (Solis BioDyne, Tartu, Estonia), 10 µM of each primer and approximately 20 ng template DNA. PCR was performed with an initial denaturation at 94°C for 5 min followed by 34 cycles of 94°C for 1 min, 55°C for 1 min and 72°C for 1 min 30 s and a final elongation step of 72°C for 5 min. After checking the quality of the PCR products by separation on a 1.5% agarose gel the replicates were pooled and purified using an E.Z.N.A. Cycle-Pure Kit (Omega Bio-Tek, Norcross, GA, USA). Cloning was done with the pGEM-T Vector System (Promega, Mannheim, Germany) and *Escherichia coli* JM109 according to the manufacturer’s instructions. Approximately 32 clones per library were screened by PCR re-amplification of the insert using M13F and M13R primers and the following PCR-conditions: 95°C for 5 min, 32 cycles of 95°C for 40 s, 54°C for 30 s and 72°C for 60 s then a final elongation step of 72°C for 10 min. Insert re-amplicons were purified with ExoSAP-IT (USB Corporation, Cleveland, OH, USA) then used in cycle sequencing with M13F as a sequencing primer and a Big Dye Terminator Cycle Sequencing Reaction Kit v.3.1 (Applied Biosystems, Foster City, CA, USA). After an ethanol precipitation sequencing was completed using an ABI 3730xl DNA Analyzer (Applied Biosystems). PolF/R primer residues were trimmed using Sequencher 4.10 (Genecodes, Ann Arbor, MI, USA). Amplified sequences were identified by BLASTn queries against NCBI GenBank using standard settings but targeting only the bacteria division. New nucleotide sequences and their MOTU (molecular operational taxonomic unit) assignments are available under accession numbers HF559482-HF560561.

### Compilation of a *nifH* Database

We have obtained, through importing into BOSQUE v. 1.81. [33], the GenBank flatfiles of all nucleic acid accession numbers contained in the ARB database provided online by the Zehr Marine Microbiology lab, University of California at Santa Cruz (updated February 17, 2012) and sent by the Buckley lab (corresponding to Gaby and Buckley [24]). Sequences that were shorter than 100 bases or of apparently non-bacterial origin were immediately excluded. Sequences that were either longer than 10,000 bases or originated from whole genome sequencing were also excluded due to difficulties in aligning multiple sequences of widely varying lengths. A local BLASTn search against our own sequences and preliminary alignment attempts on the MAFFT v. 6 server, accessed through logging in at www.bioportal.uio.no [34]–[36] were used to identify sequences for removal as well as those requiring reverse complement conversion. Another BLASTn search of our own sequences against the entire DDBJ database identified another set of *nifH* sequences to add to the growing *nifH* compilation. Then, duplicates arising from the different sources and all PolF and PolR primer annealing sites were removed while maintaining all sequence data in BioEdit v. 7.0.9.0 [37]. After using UCHIME [38] in USEARCH v. 6.0 [39] in de-novo and cross-wise modes (our sequences vs. external sequences) we flagged potential chimeras in both our wood-derived sequences and sequences from the public databases. Information from the GenBank files pertaining to origin and ecology was appended to the FASTA file headers of all hitherto retained sequences, using the Ecobyte Replace Text v. 2.2 program. The dataset of 25,303 previously published sequences together with the novel dead wood *nifH* sequences were submitted to CD-HIT [40] via www.bioportal.uio.no using a 97% cutoff threshold for building MOTUs. MOTU membership was appended to the FASTA headers of all sequences.

### Protein Alignment and Phylogenetic Analyses

We found it impossible to align even a subset of the data at the nucleotide level with confidence, thus following several other authors [41]–[43] we performed phylogenetic analyses and structure comparisons only on predicted proteins. We opted to minimize the large dataset for tractability, even for computationally intensive phylogenetic analyses, by: a) using only amino acid sequences, thus also allowing comparisons at the protein level and assortment into groups described in the literature; b) using a consensus protein sequence per MOTU rather than arbitrarily choosing reference protein sequences for non-singletons; c) representing only the MOTUs with contributions from wood-derived *nifH* and the largest overall MOTU. All new sequences and those of MOTU1 were imported again into Sequencher. The sequences known to be part of a particular MOTU were then re-assembled into a contig while singleton sequences remained separate, using amended FASTA headers to allow for automated contig building. In each contig or separate sequence we then displayed all three potential open reading frames and for ones without premature stop codon used BLASTp to find the one matching *nifH* in the public databases. Next we exported from the correct reading frame the consensus amino acid sequence for non-singleton MOTUs ( = contigs) or individual amino acid sequence for singletons. For MOTUs of mixed origin (containing our sequences and sequences from the data compiled from other sources), one consensus protein sequence or individual sequence (if there was only one) from our data was exported, and a consensus sequence or individual sequence (if one) from the remainder. For the largest group, MOTU1, which did not contain any of our sequences, this was done analogously, but separately exporting an individual sequence deviating from all others by a single amino acid. All protein sequences exported in this manner were then aligned using KALIGN2 [44] (www.ebi.ac.uk/Tools/msa/kalign/) and slightly modified manually at such indel positions where visual inspection suggested that alignment columns have been erroneously offset. Ultimately the alignment contained 111 columns. We used the 7-residue-sliding window hydrophobicity scheme in KALIGNVU [45], applying the Kyte-Doolittle method [46] to explore variability in the proteins and the WebLogo Server [47], which calculates amino acid frequency and entropy across each alignment column. Comparability to findings of other studies was maintained by using the information in the ARB file from the Zehr lab on the group memberships according to Raymond et al. [42] for the previously published sequences represented in the phylogenetic alignment and the near-BLASTn hits. Data were converted to NEXUS format using ALTER [48]. ProtTest 2 at webserver darwin.uvigo.es [49] (with PHYML and a BIONJ starting tree [50], [51]) was used to select the best model of protein sequence evolution by the Akaike Information Criterion (AIC). Hence, we used PhyloBayes v. 3.3 [52] for Bayesian inference, with commands “-nchain 2 100 0.3 50 -wag -s -dgam 6”, i.e. the WAG+Γ model chosen after ProtTest analysis (best model according to AIC: LG+I+Γ, not available in chosen programs). After examining the likelihood trace file, MESQUITE v. 2.74 [53] was used to generate one 50% majority rule consensus tree from 13,500 trees, 6,750 each after 250 burn-in trees from the two Markov chains, both leveling around ln L =  –4,000. A parameter file for GARLI [54] was generated in CIPRES v. 3.2 [55] by the GARLI.conf Creator, then we performed GARLI v. 2.0 100 replicate ML bootstrapping with WAG+Γ+I (parameters estimated) in CIPRES on the XSEDE beta server. Again, a 50% majority rule consensus tree was generated in MESQUITE from the 100 best trees of the bootstrap replicates. 100-replicate MP bootstrapping was performed in MEGA5 [56] with all gap-containing alignment columns considered, using close-neighbor-interchange (CNI) heuristics and saving a condensed consensus tree with MP bootstrap threshold of 50. The unrooted phylogenetic trees were displayed, converted, compared and edited by a number of tree editors as suitable for the output type of tree file, most notably DensiTree v. 2.01 [57] to look for conflicts between phylogenetic methods and TreeGraph2 v. 2.0.47-206 [58] to highlight tips based on the information contained in extended labels. Final combination of the Bayesian phylogenetic consensus tree with support values, clade labeling and alignment variability was done using Corel PhotoPaint X3 v. 13 (Corel, Ottawa, ON, Canada). Conflicting phylogenetic signals in the protein alignment were also assessed using SplitsTree v. 4.12.3 [59] with the model WAG+Γ (Γ shape 0.521) +I (proportion of invariable sites 0.094) in the ProteinMLdist method, producing a neighbor-network reticulogram. The entire unaligned dataset is available upon request and the protein alignment is stored in NEXUS format as File S1.

### Statistical Analysis

The effects of tree species, decay stage and forest management type on the *nifH* MOTU community structure in logs were analyzed by perMANOVA using the vegan package in R. Multivariate regression trees (MRT) [60] were subsequently applied to describe and display the relationships of the MOTUs with the independent variables by repeatedly splitting the data based on Euclidean distances. Data were then visualized in Principal Component Analysis (PCA) biplots of the group means from the prior MRT. Intersect correlations were calculated for each axis. Axes with strong correlations (>0.8) potentially account for significant between-group variation. Both PCA and MRT were performed using the mvpart package v. 1.4.0 in R. The effects of C and N content per density unit, diversity of *nifH* MOTUs and decay classes were assessed using multiple regressions with stepwise backward selection. C and N content data were log-transformed to meet the assumptions of normality. The significance and importance of the independent variables for the model were tested using ANOVA. Polynomial and linear regression were applied to display the important factors on the fructification of fungi. ANOVAs were conducted to assess the significance of the effects.

We used EcoSim [61] to test for non-random co-occurrence patterns based on presence-absence species distribution, by calculating C-scores [62] and checkerboard indices, which respectively evaluate the tendency of species not to co-occur and indicate the number of species pairs that never co-occur; using the default settings. Furthermore, we tested for non-random associations between pairs of *nifH* MOTUs and sporocarps using the PAIRS program [63]. Only fungal species and *nifH* MOTUs present in at least three samples were evaluated. A total of 100 random matrices were obtained to generate C-scores using the fixed row and fixed column constraints algorithm. Significant species under-dispersion or over-dispersion (at the 5% probability level) is indicated by Z-transformed scores (observed C-score - expected C-score) above 1.96 or below -1.96 [64]. Cytoscape [65] was used to visualize the correlations generated through PAIRS, keeping only network edges involving at least one *nifH* MOTU.

## Results

### PCR Clone Library Analysis and Clustering

**Figure 1:**
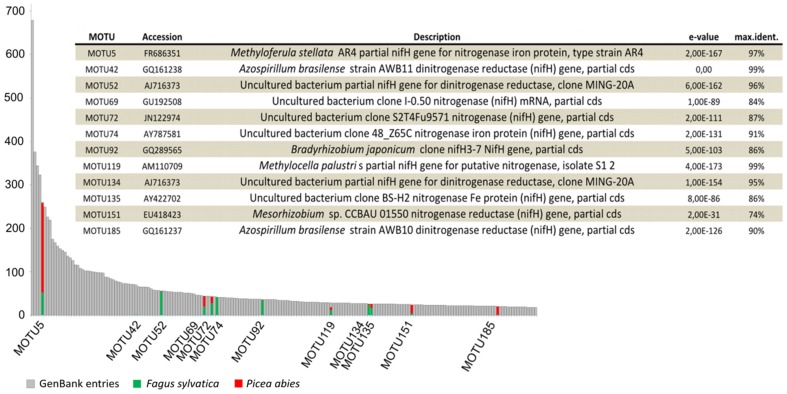
Rank abundance chart displaying the distribution of the 12 most abundant *nifH* MOTUs derived from the dead wood dataset within the compiled *nifH* dataset comprising 26,383 sequences. Only the 200 largest MOTUs are shown due to space limitations. Colored bars indicate dead wood tree species (green, *Fagus sylvatica*; red, *Picea abies*). The inserted table lists the best BLASTn hit reference sequences in NCBI Genbank for the same 12 most abundant wood-derived MOTUs from our study.

In total, 1,080 sequences were obtained by sequencing the PCR clone library derived from the dead wood samples. They were subsequently incorporated into a *nifH* compilation dataset of 25,303 sequences from GenBank, thus enlarging it to 26,383 sequences. Clustering of the sequences using CD-HIT resulted in 7,730 MOTUs of which 5,130 appeared as singletons. Among the total MOTU set, the 1,080 sequences obtained from dead wood clustered into 176 MOTUs of which 70 were singletons. Only eight of these 176 MOTUs included both sequences detected in the logs and sequences present in the 25,303 compiled GenBank entries. The rank abundance of the 200 largest MOTUs encompassing the 12 most abundant MOTUs from the dead wood samples coupled with the nearest BLAST hits from GenBank is presented in Fig. 1 and its inserted table. MOTU5, the fifth largest MOTU (Fig. 1), consisted of 260 sequences, 206 of which were obtained from *P. abies*, 52 from *F. sylvatica* logs and two from GenBank entries identified as *Methyloferula stellata* strains isolated from peat. MOTU42, the second largest MOTU including sequences from this study (Fig. 1), consisted of one sequence type revealed from dead wood together with 69 sequences from GenBank identified as *Azospirillum brasilense*, isolated from various sources (wheat rhizosphere, paddy soils, sea grasses). The most abundant MOTU1 comprised 679 GenBank sequences exclusively obtained from assays of marine samples and none were found in the analyzed logs. Roughly 11% of the 176 MOTUs could be assigned to Rhizobiales at ≥97% similiarity percentage through BLASTn against GenBank (Table S1). At a threshold of ≥90% 65 MOTUs were identified as deriving from Rhizobiales while another 15 MOTUs were associatied to dinitrogenase reductase genes from orders Rhodocyclales, Pseudomonadales, Rhodospirillales, Sphingomonadales and Burkholderiales. The majority of the MOTUs could only be identified as *nifH* associated to uncultured bacteria (Fig. 1).

**Figure 2:**
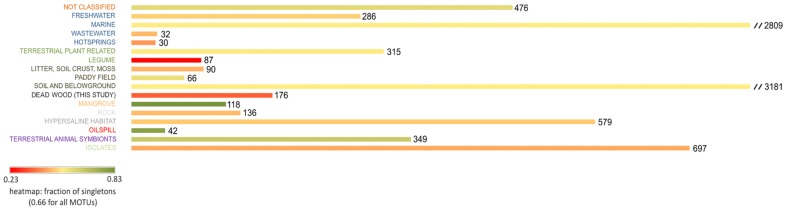
Distribution of the 7,730 *nifH* MOTUs according to the environments where they have been detected, and whether described as originating from an isolate in GenBank. 168 of the 176 MOTUs derived from this dead wood study have been exclusively identified in wood samples. The integrated heatmap displays proportions of rare sequence types (singletons).

As we assigned the 25,303 sequence types from GenBank to their source environments (according to 17 classifications, including isolates), we could analyze the distribution of all 7,730 *nifH* MOTUs in relation to habitat types (Fig. 2). The database compiled from GenBank revealed that prior to our study, no *nifH* sequences associated with dead wood had been identified, apart those associated with guts of wood-inhabiting insects (168 MOTUS of the 349 “Terrestrial Animal Symbionts”). Most sequences clustered into MOTUs assigned to the “Soil and Belowground” or “Marine” environments, with 3,181 and 2,809 counts, respectively. “Hotsprings” and “Wastewater” were the environments with the fewest MOTU counts.

### Protein Phylogenetic Analysis of the Functionality of *nifH* Genes from Dead Wood

**Figure 3:**
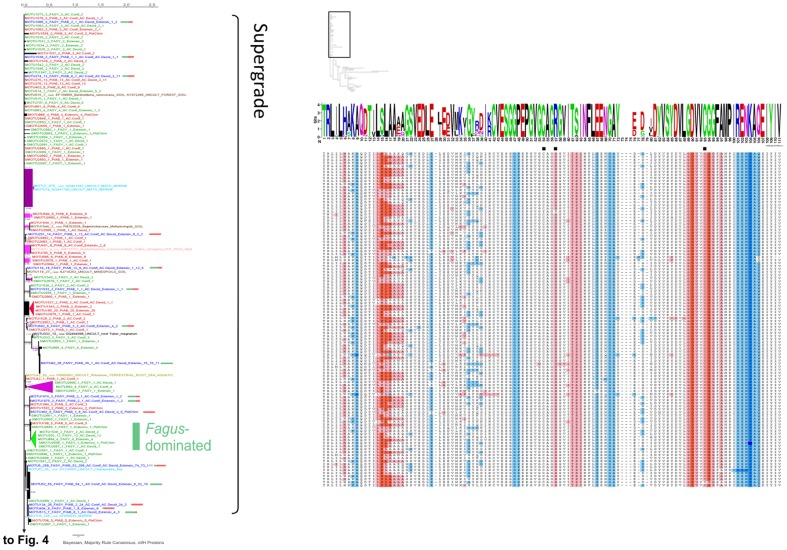
First part of three (Figs. 3, 4 and 5): Phylogenetic tree of *nifH* protein sequences. 50% majority rule consensus tree of 13,500 PhyloBayes [52] post burn-in trees, unrooted. Black values at internodes  =  Bayesian Posterior Probability (if >0.5). Pink values  =  MEGA5 [56] Maximum Parsimony (MP) bootstrap support (if >50). Green values  =  GARLI [54] Maximum Likelihood bootstrap support (if >50). Terminal triangles represent monophyletic clades with MOTUs solely of one tree species origin, collapsed but keeping the internal distance (substitutions per site, see scale bar), in light pink  =  50–79 MP bootstrap support, dark pink  =  80–100 MP bootstrap support. Green color indicates MOTUs solely from *Fagus* origin, red color *Picea* origin and dark blue color mixed origin (with bars showing ratio of [green] vs. [red]). Terminal labels with sequences from this study: MOTU ID (SMOTU  =  singleton MOTU), total number of sequences, FASY  =  from *Fagus*, PIAB  =  from *Picea*, followed by number of sequences in the same order, then forest management type(s) (AC.Conif  =  managed spruce forests, AC.Decid  =  managed beech forests, Extensiv  =  extensively managed beech forests) and number of sequences in same order. Terminal labels with sequences from other sources: near BLAST hit, summary of ecological data of sequences in that MOTU. MOTUs that contain nucleotide sequences flagged as potential chimeras appear in italics and with the term PotChim (only present in Fig. 3, Supergrade). The width of visible terminal branches represents the number of sequences (size correct up to 10 sequences). To the right, amino acid sequence logos and Kyte-Doolittle hydophobicity alignments for labeled nodes on the tree. The small tree shape (based on screenshot from Archaeopteryx v.0.972 [66]) shows the position within the complete phylogenetic tree.

**Figure 4:**
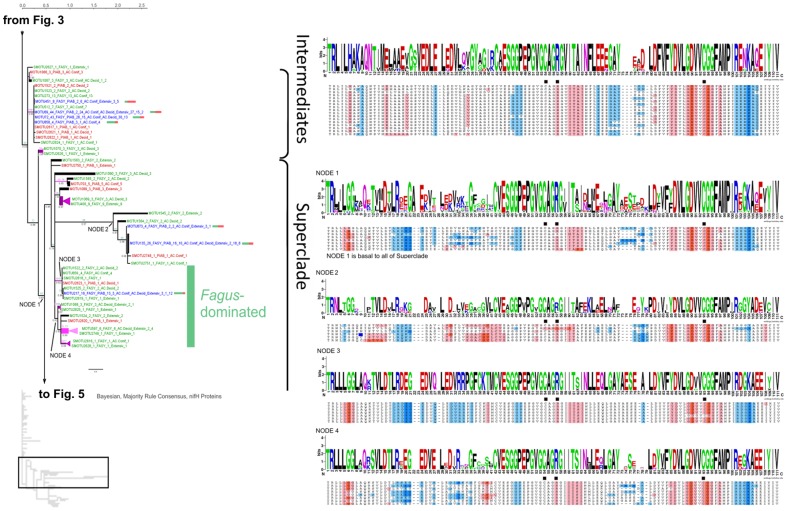
Second part of three (Figs. 3, 4 and 5): Phylogenetic tree of *nifH* protein sequences. 50% majority rule consensus tree of 13,500 PhyloBayes [52] post burn-in trees, unrooted. Black values at internodes  =  Bayesian Posterior Probability (if >0.5). Pink values  =  MEGA5 [56] Maximum Parsimony (MP) bootstrap support (if >50). Green values  =  GARLI [54] Maximum Likelihood bootstrap support (if >50). Terminal triangles represent monophyletic clades with MOTUs solely of one tree species origin, collapsed but keeping the internal distance (substitutions per site, see scale bar), in light pink  =  50–79 MP bootstrap support, dark pink  =  80–100 MP bootstrap support. Green color indicates MOTUs solely from *Fagus* origin, red color *Picea* origin and dark blue color mixed origin (with bars showing ratio of [green] vs. [red]). Terminal labels with sequences from this study: MOTU ID (SMOTU  =  singleton MOTU), total number of sequences, FASY  =  from *Fagus*, PIAB  =  from *Picea*, followed by number of sequences in the same order, then forest management type(s) (AC.Conif  =  managed spruce forests, AC.Decid  =  managed beech forests, Extensiv  =  extensively managed beech forests) and number of sequences in same order. Terminal labels with sequences from other sources: near BLAST hit, summary of ecological data of sequences in that MOTU. The width of visible terminal branches represents the number of sequences (size correct up to 10 sequences). To the right, amino acid sequence logos and Kyte-Doolittle hydophobicity alignments for labeled nodes on the tree. The small tree shape (based on screenshot from Archaeopteryx v.0.972 [66]) shows the position within the complete phylogenetic tree.

**Figure 5:**
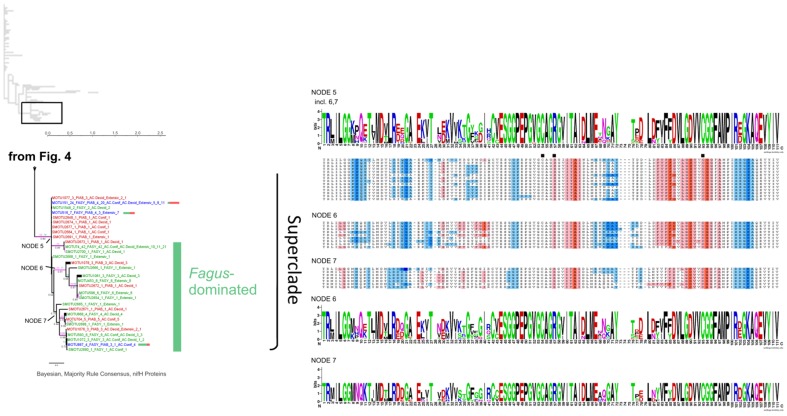
Third part of three (Figs. 3, 4 and 5): Phylogenetic tree of *nifH* protein sequences. 50% majority rule consensus tree of 13,500 PhyloBayes [52] post burn-in trees, unrooted. Black values at internodes  =  Bayesian Posterior Probability (if >0.5). Pink values  =  MEGA5 [56] Maximum Parsimony (MP) bootstrap support (if >50). Green values  =  GARLI [54] Maximum Likelihood bootstrap support (if >50). Terminal triangles represent monophyletic clades with MOTUs solely of one tree species origin, collapsed but keeping the internal distance (substitutions per site, see scale bar), in light pink  =  50–79 MP bootstrap support, dark pink  =  80–100 MP bootstrap support. Green color indicates MOTUs solely from *Fagus* origin, red color *Picea* origin and dark blue color mixed origin (with bars showing ratio of [green] vs. [red]). Terminal labels with sequences from this study: MOTU ID (SMOTU  =  singleton MOTU), total number of sequences, FASY  =  from *Fagus*, PIAB  =  from *Picea*, followed by number of sequences in the same order, then forest management type(s) (AC.Conif  =  managed spruce forests, AC.Decid  =  managed beech forests, Extensiv  =  extensively managed beech forests) and number of sequences in same order. Terminal labels with sequences from other sources: near BLAST hit, summary of ecological data of sequences in that MOTU. The width of visible terminal branches represents the number of sequences (size correct up to 10 sequences). To the right, amino acid sequence logos and Kyte-Doolittle hydophobicity alignments for labeled nodes on the tree. The small tree shape (based on screenshot from Archaeopteryx v.0.972 [66]) shows the position within the complete phylogenetic tree.

We performed a protein phylogenetic study on the 176 MOTUs corresponding to all sequences we detected in dead wood plus the most abundant MOTU (MOTU1), which did not include *nifH* sequences from our dead wood. In this part of the study a consensus protein sequence was exported from Sequencher for each non-singleton MOTU. Singleton sequences and a deviant within MOTU1 were translated into protein sequences. From the eight dead wood MOTUs that also contained sequences from other studies found in distinct habitats (MOTUs 5, 42, 52, 119, 332, 401, 515 and 1544), one translated protein sequence not corresponding to the wood habitat was exported as an individual sequence (if one) or consensus sequence (if more than one) and added to the analyses. In total, 186 protein sequence types (also annotated with source tree species if derived from dead wood) were used for phylogenetic calculations. Phylogenetic analyses revealed that many sequences assemble in a long comb, with short branches, henceforth called “Supergrade” (Fig. 3, compare Figs. 4, 5). All protein motifs within that clade appeared to be similar and exclusively correspond to the group called Group I by Raymond et al. [41]. The part of the tree designated “Superclade” was better resolved and was dissected into seven nodes (1–7) (Figs. 4, 5). Nodes 3/4 and 5 to 7 (Figs. 4, 5) were exclusively populated by sequences of Groups II and III [41], respectively, mainly derived from *F. sylvatica* logs. A notable exception was node 2, the base of a long branch, containing sequences of Group IV, previously characterized as less certain to be really involved in nitrogen fixation. The transitional part of the tree between the poorly and relatively well resolved branches, designated as “Intermediates”, hosts members of *nifH* Groups I and III. The protein phylogeny did not show a full separation of *nifH* sequences by tree species, indeed the highly conserved protein motifs in the Supergrade appeared to contain almost equal numbers of sequences from each tree species. Only the MOTUs that assembled in nodes 3–7 of the Superclade were predominantly from *Fagus sylvatica*. Protein sequences at labeled nodes showed distinct differences in amino acid composition as displayed in the alignments visualized by using KALIGNVU [44] and WebLogo [46] (Figs. 3, 4, 5). All protein sequence types contained the iron sulfur coordinating cysteines that are marked with black squares in Figs. 3, 4, 5. Splitstree analysis (Fig. S2) also divided the phylogenetic tree into relatively well and poorly resolved parts. The broader tree structure of the reticulogram (Fig. S2) is the same as in Figs. 3, 4, 5, but better shows phylogenetic distances because it is given as unrooted network. 53 potential chimeras that appeared in 9 MOTUs were only detected within the Supergrade, which has weak phylogenetic resolution.

### *nifH* Sequence Diversity

**Figure 6:**
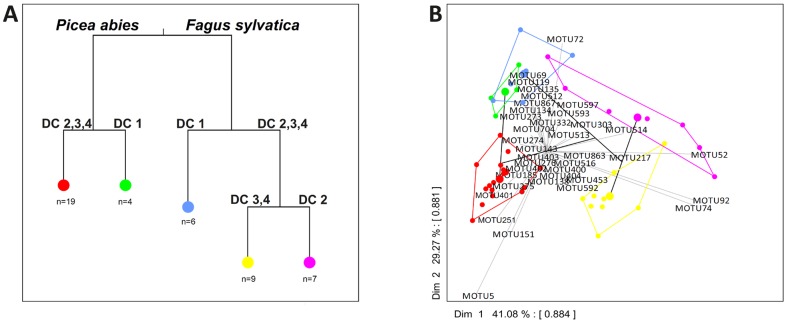
*nifH* community structure A: Multivariate regression tree of *nifH* MOTU community composition estimated from sequences of the clone library obtained from dead wood of *Picea abies* and *Fagus sylvatica*. Analyses were conducted for different decay classes, based on the remaining mass per dead wood log after decay using k-means cluster analysis. B: Principal Component Analysis biplots of the group means of the multivariate regression tree. The larger circles (per color) represent the multivariate group means, the individual logs are denoted by smaller circles, with matching colors and designation to Fig. 6A. The identity of selected MOTUs with characteristic discriminatory loading is specified. Each MOTU label is located at its weighted mean from the group means. Intersect correlation is given in brackets.

Analysis of the richness and community distribution of the 176 *nifH* MOTUs revealed 3 to 14 different MOTUs per dead wood log, and significantly higher richness in *F. sylvatica* than in *P. abies* logs (Fig. S3A; p = 0.028). PerMANOVA analysis revealed that both tree species and decay class significantly explained the variation of the *nifH* community on dead wood (p = 0.001), but not forest management type (Table 1). Multivariate regression analysis clearly separated the *nifH* communities according to tree species (Fig. 6A). Within dead wood of the different species, the decay class was the dominant factor for the separation. Notably the communities in *Fagus sylvatica* logs and *Picea abies* logs of the least decomposed stage 1 were clearly distinct from the communities of more decayed dead wood logs. On *F. sylvatica* logs a broader branching of decay stage 2 from stages 3 and 4 explained the separation of *nifH* community structure. The first principle component (PC) of the PCA (Fig. 6B) explained roughly 41% of the total variation, and mainly separated MOTUs associated with *F. sylvatica* logs of intermediate decay stage 2 (driven by MOTU52, the fourth most abundant in the dataset) and decay stages 3 and 4 from sequences associated with *P. abies* logs of decomposition stage 2. The second PC explained nearly 30% of the community variation and separated the *nifH* sequence types detected on *F. sylvatica* logs of decay classes 1 and *P. abies* logs as well as MOTU5, the most abundant MOTU in this dataset (with 258 sequences, 206 of them from *P. abies* dead wood).

**Table 1:** Results of perMANOVA analysis of Bray-Curtis dissimilarities in *nifH* MOTU community structure in relation to tree species, decay class (based on remaining mass after decay) and management type and their interactions, Df  =  degrees of freedom; SS  =  sum of squares; MS  =  mean sum of squares; Pseudo-F  =  F value by permutation, boldface indicates statistical significance at p<0.05, p-values based on 999 permutations (lowest p-value possible is 0.001).

	Df	SS	MS	*F*	*R^2^*	*P*
Tree species	1	1.376	1.376	3.929	0.079	**0.001**
Decay class	1	1.073	1.073	3.064	0.061	**0.001**
Management type	2	0.757	0.378	1.08	0.043	0.322
Tree species x Decay class	1	0.589	0.589	1.681	0.034	**0.024**
Tree species x Management type	2	0.575	0.287	0.821	0.033	0.847
Decay class x Management type	2	0.712	0.356	1.016	0.041	0.447
Tree species x Decay class x Management type	2	0.84	0.42	1.198	0.048	0.157
Residuals	33	11.56	0.35	0.661		
Total	44	17.481	1			

### Fungal Diversity and Influencing Factors

**Figure 7:**
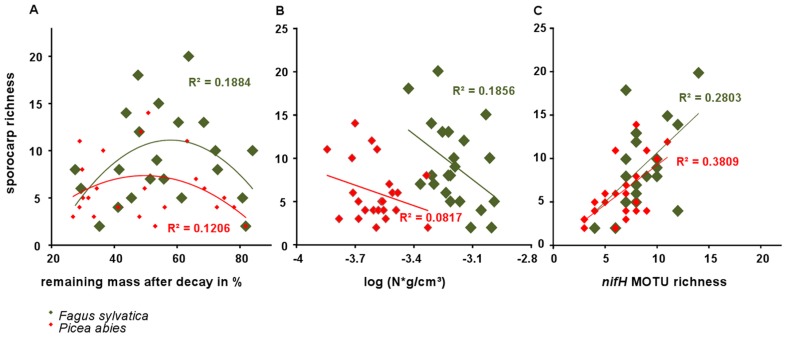
Interrelations between sporocarp richness and remaining mass after decay in %, *nifH* MOTU richness and log-transformed nitrogen content per density unit (N (g/cm^3^))(A, B, C). The figure displays interrelations separately per dead wood species.

In total 158 fungal species were detected on the dead wood logs and 131 (83%) of them could be identified to the species level (Table S2). Fungal species richness ranged from 2 to 20 observed species on *Fagus* logs and from 2 to 14 on logs of *Picea abies* (Fig. S4). Mean species richness was significantly higher (ANOVA, p = 0.027) on *Fagus sylvatica* (9.14 ± 1.01) versus *Picea abies* (6.04 ± 0.71) (Fig. S4). We also observed a wider Basidiomycota to Ascomycota ratio on *Picea abies*. Barely 2% of the detected species belonged to the phylum Ascomycota, while they accounted for 40.3 % of all taxa being observed on *Fagus sylvatica* (Fig. S5). ANOVA of factors affecting sporocarp richness based on multiple regression analyses also revealed that tree species significantly (p = 0.005) affected the fungal diversity (Table 2), as well as N content (p = 0.003), decay class (based on the remaining mass after decay; p = 0.011), and *nifH* MOTU richness (p<0.001). In contrast, C content did not influence the diversity of sporocarps on dead wood (p = 0.896). Polynomial and linear regressions were performed separately for the *F. sylvatica* and *P. abies* logs to avoid redundancy in the analyses, since tree species had the strongest effects on chemical constitution and *nifH* MOTU richness, as displayed in Figs. S3 and S8. Fungal diversity was related to the decay class. The highest number of sporocarps was observed on logs of intermediate stages of mass loss (Fig. 7A *F. sylvatica* R^2^ = 0.1884, p<0.05, *P. abies* R^2^ = 0.1206, p = 0.14), which was significant on logs of *Fagus*. We also observed a negative correlation between N content and sporocarp richness (Fig. 7B; *F. sylvatica* R^2^ = 0.1856, p = 0.04) that was significant for *F. sylvatica* logs. There was no correlation between remaining mass after decay and *nifH* MOTU richness (R^2^ = 0.0701, p = 0.28, Fig. S6A, *F. sylvatica* only), but polynomial regression revealed that the number of MOTUs peaked at 54.9% water content (R^2^ = 0.268, p<0.02, Fig. S6B), which in turn significantly correlates with the intermediate decay stages (R^2^ = 0.7396, p<0.001, Fig. S6C). Furthermore, we also observed a significantly positive correlation between diversity of *nifH* MOTUs and sporocarp species richness on logs of both tree species (Fig. 7C: *F. sylvatica* R^2^ = 0.2803, p = 0.011; *P. abies* R^2^ = 0.3809, p = 0.0017).

**Table 2:** ANOVA table of effects of the indicated factors on fungal fructification ability.

	Sporocarp richness				
	Df	SS	MS	*F*	*P*
Tree species	1	1.784	1.784	8.812	**0.005**
log (Ng/cm^3^)	1	2.015	2.015	9.952	**0.003**
log (Cg/cm^3^)	1	0.004	0.004	0.017	0.896
Decay class (remaining mass after decay)	1	1.457	1.457	7.198	**0.011**
*nifH* MOTU richness	1	3.32	3.32	16.399	**<0.001**
Residuals	39	7.896	0.203		

Complete model summary representing *R^2^, F, P* statistics. Abbreviations of the depicted ANOVA table Df  =  degrees of freedom, SS  =  sum of squares, MS  =  mean sum of squares. The summary model is as follows: *R^2^*, *F*, and *p* were 0.5208,

8.476 and <0.001 (significant), respectively. Boldface indicates statistical significance.

### Co-occurrence Patterns

**Figure 8:**
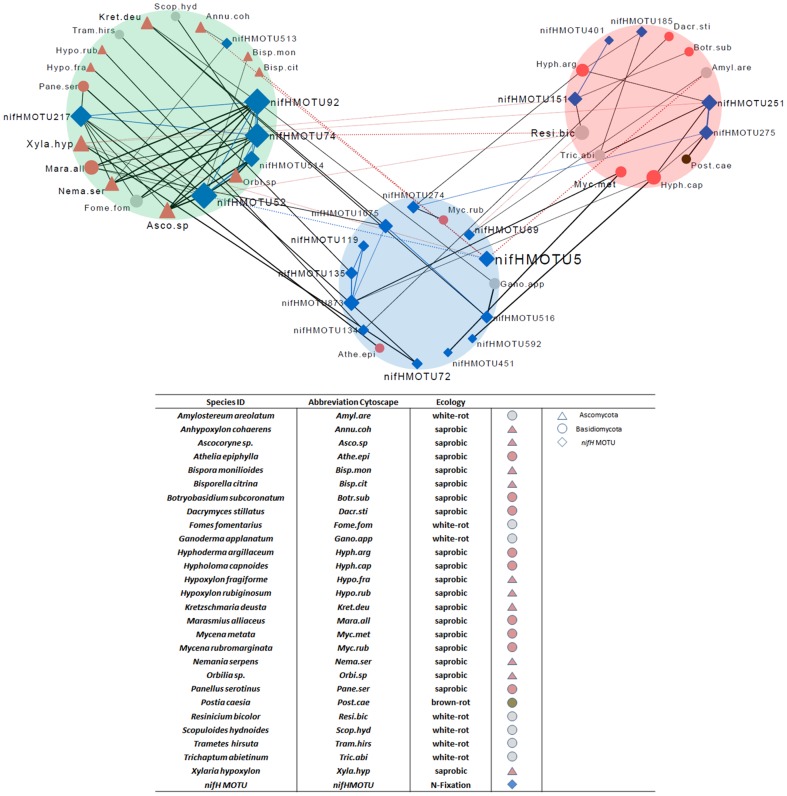
Network organized around 23 *nifH* MOTUs and 27 fungal species (abbreviations according to the legend). Fungi (sporocarps) and *nifH* MOTUs serve as connected nodes, solid lines display co-occurrence patterns (Z-Score < -1.96) and dotted lines avoidance patterns. Edge widths display significance levels from thinnest  =  0.049 to thickest  =  0.0017. Differently shaped and colored nodes/ hubs display taxonomic differences on phylum level and their ecological role in wood decay. Subnetworks are grouped by tree species, and colored background circles indicate affiliations of included taxa to substrate dead wood species (green  =  *Fagus* affiliated, red  =  *Picea* affiliated, blue  =  unaffiliated “Generalists”).

C-score and checkerboard analyses revealed structured, non-random associations between bacterial *nifH* and fungal sporocarps. Observed C-score and checkerboard index values (20.1712 and 940.0, respectively; Fig. S7) were both significantly higher than the expected values from randomized datasets (C-score = 19.80343, p<0.0001 and checkerboard = 841.0292, p<0.0001). The pairwise relationships in this assembled community were thus further detailed using PAIRS. A null model analysis using fixed rows and columns resulted in 54 correlations among *nifH* MOTUs and sporocarps with significant Z-transformed scores above 1.96 and below -1.96. By incorporating information on preferential occurrences of fungi and *nifH* MOTUs according to dead wood species (using a threshold of 75% to classify them as either *Fagus* or *Picea* affiliated; otherwise substrate “generalists”) we identified both positive associations and avoidance patterns in the context of the two different wood-species substrates (Fig. 8). The *Fagus* and *Picea* subnetworks entail four and two positive co-associations within *nifH* sequences, respectively. The ambiguous subnetwork of generalists contains more *nifH* than fungi, but only five positive co-associations between different *nifH*. The *Fagus* subnetwork is the largest, dominated by fungi with well-described functions as wood-decayers and mainly abundant *nifH* MOTUs. There are no positive associations bridging the *Fagus* and *Picea* subnetworks. Most associations that link either *Fagus* or *Picea* with the ambiguous subnetwork are positive.

Network Analyzer [66] was used to identify hub nodes from their degree distribution. The 50 nodes were connected to 3.16 neighbors on average. *nifH* MOTUs 92, 74, 217 and 52 (which are highly connected to other nodes: >10 degrees) could be identified as hubs, presumed to be stable towards random node removal from the network. Ten *nifH* MOTUs in the ambiguous subnetwork (meaning they occurred more evenly on both *Fagus* and *Picea*) also show positive associations with fungi. For example, *nifH* MOTU72 co-occurs with Xylariales *Hypoxylon fragiforme* and *Xylaria hyopoxylon*. *nifH* MOTU5 (detected on 11 *Fagus* and 19 *Picea* logs) only avoids Xylariales *Annulohypoxylon cohaerens* and *Xylaria hyopoxylon* as well as the Helotiales *Bisporella citrina* (all known nearly exclusively from *Fagus* logs). Six *nifH* MOTUs (72, 274, 451, 592, 873 and 1075) significantly co-occur with fungal species that were solely present on either dead wood species.

## Discussion

### Characterization and Diversity of *nifH* Sequences in Dead Wood

Gaby and Buckley [24] stated that the diversity of *nifH* is not evenly distributed across various environments and that further investigations are required to understand the links and specificity of diazotrophic communities to their substrates. Our approach led to the discovery of a rich diversity of novel *nifH* sequences in hitherto unexplored dead wood substrate that corresponds to presumably active *nifH*. Assigning the 1,080 new sequences to MOTUs with a large backing compilation of previously published *nifH* sequences from public databases allowed us to examine specificities of the *nifH* pools in source environments. The finding that only eight among the 176 (containing sequences from this study) included sequences derived from other environments indicates that dead wood is a specific substrate for N-fixing bacteria. The proportion of singletons (39%) within this dead wood dataset of 176 MOTUs was below the average value (66%) for all MOTUs derived from 17 source classifications (16 environments plus isolates from cultures), as displayed in Fig. 2. This can be explained by the proximity of the investigated logs, all of which originated from nine plots at a single experimental site in Germany, while the MOTUs retrieved from GenBank were from geographically widely distant sources. MOTU composition mainly differed between the tree species. While 63 and 87 MOTUs were exclusively detected in *P. abies* and *F. sylvatica* dead wood, respectively, only 26 MOTUs were common to both tree species. BLASTp and additional comparison with the Zehr database indicated that almost all detected *nifH* sequences were members of *nifH* Groups I, II and III designated by Raymond et al. [42], with the notable exception of node 2 at the base of a long branch. These groups are thought to be functional *nifH* genes. Accordingly, the amino acid sequence around the cysteines required for formation of Fe_4_S_4_ clusters at alignment positions 54 and 93 (Figs. 3, 4, 5) also correspond to these groups, as depicted in Fig. 6 of the cited article [42]. Collectively, the evidence suggests that almost all *nifH* sequences detected in this study encode real dinitrogen reductase. MOTUs that assembled in the Supergrade of the phylogenetic tree exclusively correspond to *nifH* Group I, which primarily originate from Cyanobacteria and Proteobacteria [42]. The latter produce dinitrogenase reductases with Fe_4_S_4_ clusters that are inactivated and inhibited by O_2_ [67]. We found that roughly 78% of our 1,080 sequences belong to Group I, which also was the most numerous group according to Wang et al. [26]. As wood decay advances, water content is likely to rise and oxygen levels to fall, at least seasonally [14], and higher N-fixation rates have been detected under anaerobic than aerobic conditions in fallen tree boles of *Pseudotsuga menziesii* [68]. Oxygen depletion in more water-logged or decayed logs could allow the activity of oxygen-sensitive *nifH* genes from Group I. Accordingly, the transitional part of the tree between the poorly and relatively well resolved branches, designated “Intermediates”, hosts members of both *nifH* Groups I and III. Only the six MOTUs in node 2 contained sequences that point to membership of *nifH* Group IV. The 36 dead wood *nifH* sequences (from only eight logs) included in these six MOTUs have GenBank matches with at most 69–86% identity according to BLASTn searches. The further resolved nodes 3, 4 and 5 to 7 were exclusively comprised of sequences affiliated with Groups II and III, respectively. MOTUs that clustered in these branches were mainly derived from *F. sylvatica*, possibly due to differences in the chemical constitution of the two species’ wood. Wazny and Wazny [69] measured micronutrient concentrations in 34 tree species and found vanadium concentrations were highest in *Pinus nigra* and *P. abies*, which seems inconsistent with our finding of Group III sequences (alternative nitrogenases, in which for instance vanadium replaces molybdenium) mainly in dead *F. sylvatica* wood, but should be subject to further investigation.

### Correlations of *nifH* Community Structure with Environmental Settings

Phylogenetic reconstruction revealed that tree species strongly influence *nifH* diversity, a conclusion supported by the multivariate statistics and network analyses. Only 26 MOTUs were detected in both *F. sylvatica* and *P. abies* logs, and MOTU richness was significantly higher in *F. sylvatica* logs (Fig. S3A). Differences in chemical and structural parameters of the species’ wood could explain this pattern. For example, wood density and C content (g*cm^-^^3^) were both significantly higher in *Fagus sylvatica* logs (p<0.001, Fig. S8). The four stages of decay, distinguished on the basis of remaining mass, also significantly separated the *nifH* community. Notably, there were significant differences between the structures of the *nifH* communities associated with *F. sylvatica* logs of initial decay stage 1 and later stages (Fig. 6A). The results for *P. abies* logs revealed similar segregation of the pools of *nifH* sequences associated with initial and advanced decay stages. Hicks et al. [70] and Spano et al. [15] reported contrasting fixation rates in coniferous wood, finding them to be highest in moderate phases of decay and later phases of decay, respectively. How well *nifH* diversity relates to actual N-fixation rates in dead wood is hence still an open question. The intensity of forest management in the different forest plots did not influence the distribution of *nifH* sequence types: the highest variations in MOTU richness were found in logs of extensively managed forests, but mean values did not differ significantly among the different management types. Brunner and Kimmins [7] proposed that the amount of available dead wood significantly affects N-fixation, finding ranges in rates from 1 to 2.1 kg*ha^−1^*year^−1^, and hence long-term N accumulation in unpolluted ecosystems. As we have no basis to estimate actual N-fixation rates in our sampled logs and correlate them with as yet unavailable data on quantities of dead wood at the study sites, we can hardly address this issue. However, Shaffer and colleagues [71] observed a decline in the *nifH* gene pool in litter of Douglas fir forests when they were clearcut, and hence proposed that the lost diversity could potentially contribute more to N-fixation in forest litter than in litter from plants that regrow in clearcuts.

### Interrelation of Fungal Fructification and N-fixing Bacteria through N-Availability

The impression that dead wood is a specific substrate whose decomposer communities harbor N-fixers is reinforced by variations in wood decay progression, fruiting body production and variations in N concentration during wood decomposition. Dead wood is a complex, heterogeneous and dynamic environment. Thus, several factors probably contribute to the presence of unique pools of presumably functional *nifH* sequences in our sampled dead wood community. Our finding of fewer singletons than in some other environments points to greater sequencing depth and spatial closeness of our samples, and the weak overlap with sequences from other environments to dead wood as a unique environment. Our hypothesis that *nifH* community structure is interrelated with fungal occurrences on dead wood was supported by positive correlations between sporocarp- and *nifH* MOTU richness (Fig. 7C). Heilmann-Clausen [10] reported that sporocarp richness was maximal in the intermediate decay stages, when N content in logs was lowest, while N-concentration continuously increased as decomposition proceeds (Fig. S9), as also observed by Volkenant [14] and Boddy and Watkinson [13]. This may reflect the high need for N in the phase of highest fungal vegetative and generative growth. A similar temporal pattern of N characteristics has been observed for Japanese *Fagus crenata* wood [72]. We propose that further N-accumulation may be due to actively N-fixing bacteria and the decline of sporocarp occurrence and sporocarp biomass during loss of wood mass. This is consistent with pioneering studies by Merrill and Cowling [73] and Larsen et al. [21], who first suggested that fungi overcome N deficiencies by interaction with N-fixing bacteria and subsequently confirmed that fixation occurs in living sporocarps, respectively. Other opportunities for N accumulation that should be considered include its release and recycling from eaten and degenerating fruiting bodies as well as spores falling back onto and into the dead wood [18], [73]. In addition, fungi that grow hyphae not only in dead wood but also in soils will likely access N pools outside dead wood and move it to places where it is needed most. Another potential link between the co-presence of both many *nifH* sequences and many fungi in dead wood is that specific bacteria may associate with specific fungi, perhaps in symbiotic or at least specialized commensal relationships. Intimate cooperation between fungi and bacteria in the process of decaying lignocellulosic material may thus be another widespread and ecologically important aspect of fungal-bacterial interaction [74]. Network analysis based on non-random co-occurrence patterns revealed relationships between sporocaps and *nifH* MOTUs. Both C-score and checkerboard analyses indicated that bacteria and fungi co-occur less often than expected by chance. Their assemblages on dead wood were structured and clearly depend on the different tree species.

The network analysis revealed that co-associations between *nifH* MOTUs and fungi are strongly dependent on substrate qualities. Interpretation of these relationships is not straightforward, which is due to the lack of validation of real N-fixation in our samples. However, we can assume that these positive correlations among taxa are due to cross-feeding, co-colonization, niche overlaps [75] or a combination of these possibilities. These patterns could also follow community assembly rules, as originally proposed by Diamond [76] and still debated by ecologists [77]. Distributions of fungi and bacteria across various environments are determined by their dispersion and adaption mechanisms [78], [79]. The results of our study provide information that potentially N-fixing bacteria, detected by the presence of diverse *nifH* genes, are distributed across complex environments analogously to fungi. As previously mentioned, numerous studies have confirmed that biotic N-fixation occurs in dead wood and forest soils, as well as providing valuable contributions to our understanding of N-related traits and resulting ecosystem services. The dispersion and ecology of the microbial communities involved were described in 2000, but the cited study merely focused on community shifts under different forest management regimes [71]. Our investigation lacks information on real activity, but our results allowed us to detect and discern differences in the communities of N-fixing bacteria in terms of *nifH* genes in two kinds of substrates. As we included environmental information in the network analysis we were also able to identify conditions that the co-occurring species assemblages preferred or avoided. Explanations for observed patterns of *nifH* gene diversity include niche differentiations according to water content and oxygen depletion in different decomposition stages, or variations in the chemical constitution of particular tree species based on different wood decay types, resulting in variations in cellulose levels [80] and consequently ATP availability [18]. Whether the observed co-occurrence patterns result directly from bacterial-fungal interactions or from more complex, indirect interactions, remains to be elucidated.

To assess whether N newly fixed by bacteria reaches fungal fruiting bodies, labeling experiments with ^15^N are needed. The labeled substances should include N_2_ and other N containing compounds in surrounding soil as alternative N sources, as some fungi may bridge soil, litter and decaying wood, e.g., [81]. In addition, data on transcription and activity of the transcripts must be correlated with fungal biomass, diversity and enzymatic activities of lignocellulose degrading enzymes.

## Supporting Information

Figure S1 **Sampling scheme visualized using Treemap v. 3.1.0. (Macrofocus, Zurich, Switzerland) in squarified layout.** Items are grouped by management type. Treemap cell size is proportional to mass loss in % (smaller cells  =  less decayed logs) Colors represent tree species. (red  =  *Picea abies*, green  =  *Fagus sylvatica*). Numbers indicate the ID of the dead wood item.
(TIF)

Figure S2 **Splitstree reticulogram.** The three major parts of the phylogeny (compare phylogenetic tree in Figs. 3, 4, 5) are labeled here.
(TIF)

Figure S3 **Bargraphs including standard errors displaying** ***nifH*** **MOTU richness (A) and nitrogen content per density unit (B) within dead wood tree species.**
(TIF)

Figure S4 **Scatterplot displaying number ( =  richness) of fruiting fungal species per dead wood log.** Solid vertical lines display mean values of richness, dashed vertical lines median richness per tree species (green  =  *Fagus sylvatica*, red  =  *Picea abies*). Heatmapped bars to the left and right display density probability as calculated by kernel density estimation using the denstrip package in R (Jackson CH (2008) Displaying uncertainty with shading. Am Stat 62: 340-347.).
(TIF)

Figure S5 **Relative abundances (left) of Basidiomycota and Ascomycota on dead wood logs of** ***Fagus sylvatica*** **and** ***Picea abies*** **and mean number of sporocarps per tree species (right).**
(TIF)

Figure S6 **Interrelation of** ***nifH*** **MOTU richness and remaining mass after decay in % (A) and water content in % (B) and water content in % and remaining mass after decay in % (C) on logs of** ***Fagus sylvatica*** **.**
(TIF)

Figure S7 **Non-random sporocarp –** ***nifH*** **MOTU community assembly assessed by C-score distribution and Checkerboard index for observed and expected/ randomized species occurrences.**
(TIF)

Figure S8 **Boxplots including median, upper and under quartiles and whiskers displaying the interrelation of dead wood species and log transformed carbon content per density unit (A) and log transformed wood density (B).**
(TIF)

Figure S9 Effects of remaining mass after decay in % on log-transformed nitrogen content per density unit (g*cm^3^) and N concentration in g*g^-1^. Interrelations are displayed separately per wood species.
(TIF)

Table S1 **Sequence percentage identity of MOTUs taxonomically assigned through BLASTn against GenBank (uncultered/ environmental sample sequences excluded).** 19 MOTUs were assigned to Rhizobiales at a 97% similarity threshold (65 MOTUs at ≥90%). A total of 80 MOTUs were identified to genus level.
(DOCX)

Table S2 **List of sporocarps identified on the respective dead wood trees A)** ***Fagus sylvatica*** **B)** ***Picea abies.***
(DOCX)

File S1 **Protein Alignment in NEXUS Format.**
(TXT)
