# Correction: Network Analysis Reveals Ecological Links between N-Fixing Bacteria and Wood-Decaying Fungi

**DOI:** 10.1371/journal.pone.0091389

**Published:** 2014-03-14

**Authors:** The PLOS ONE Staff

Supplementary Table S1 is incorrect. Please see a corrected version of Supplementary Table S1 below.

In addition, the title and legend of Supplementary Table S1 is incorrect. The correct title and legend are:

## Supporting Information

Table S1 **Sequence percentage identity of MOTUs taxonomically assigned through BLASTn against GenBank (uncultured/ environmental sample sequences excluded).** 19 MOTUs were assigned to Rhizobiales at a 95% similarity threshold (65 MOTUs at ≥90%). A total of 80 MOTUs were identified to genus level.
(DOCX)

## 

In addition, the 10th sentence of the "PCR Clone Library Analysis and Clustering" section of the Results is incorrect. The 10th sentence should read: "Roughly 11% of the 176 MOTUs could be assigned to Rhizobiales at ≥97% similiarity percentage through BLASTn against GenBank (Table S1)."

In addition, in the 10th sentence of the "Characterization and Diversity of *nifH* Sequences in Dead Wood," the term "Fig. 6" incorrectly links to Figure 6 in the article. The term refers to a figure in another paper cited in the article.
