# Supplementary figures and images for: Network Analysis Reveals Ecological Links between N-Fixing Bacteria and Wood-Decaying Fungi
Source: PLoS One. 2014 Feb 5;9(2):e88141. doi: 10.1371/journal.pone.0088141 (PMC3914916; doi:10.1371/journal.pone.0088141)

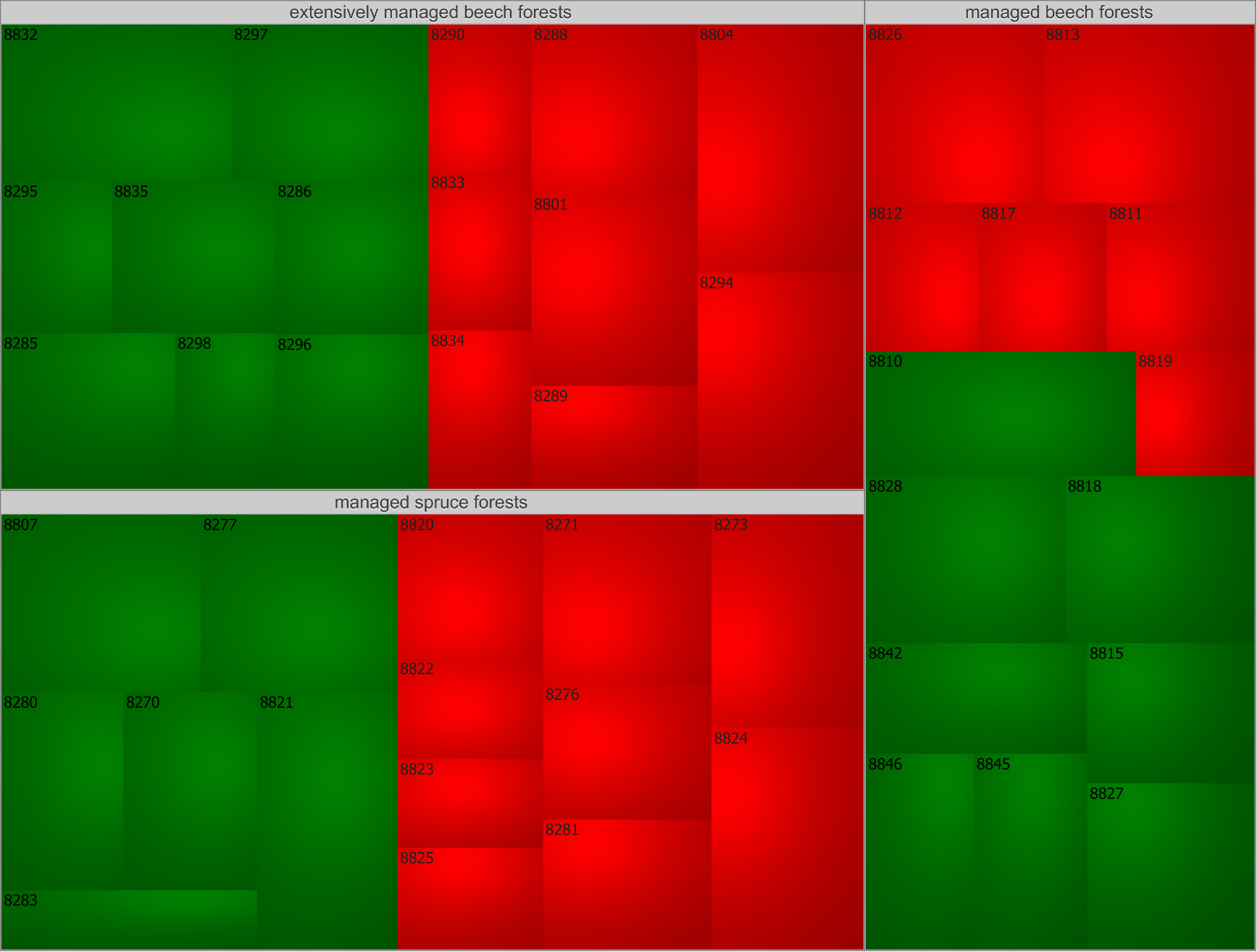

Supplement: Figure S1 — Sampling scheme visualized using Treemap v. 3.1.0. (Macrofocus, Zurich, Switzerland) in squarified layout. Items are grouped by management type. Treemap cell size is proportional to mass loss in % (smaller cells = less decayed logs) Colors represent tree species. (red = Picea abies, green = Fagus sylvatica). Numbers indicate the ID of the dead wood item. (TIF) [file pone.0088141.s001.tif]

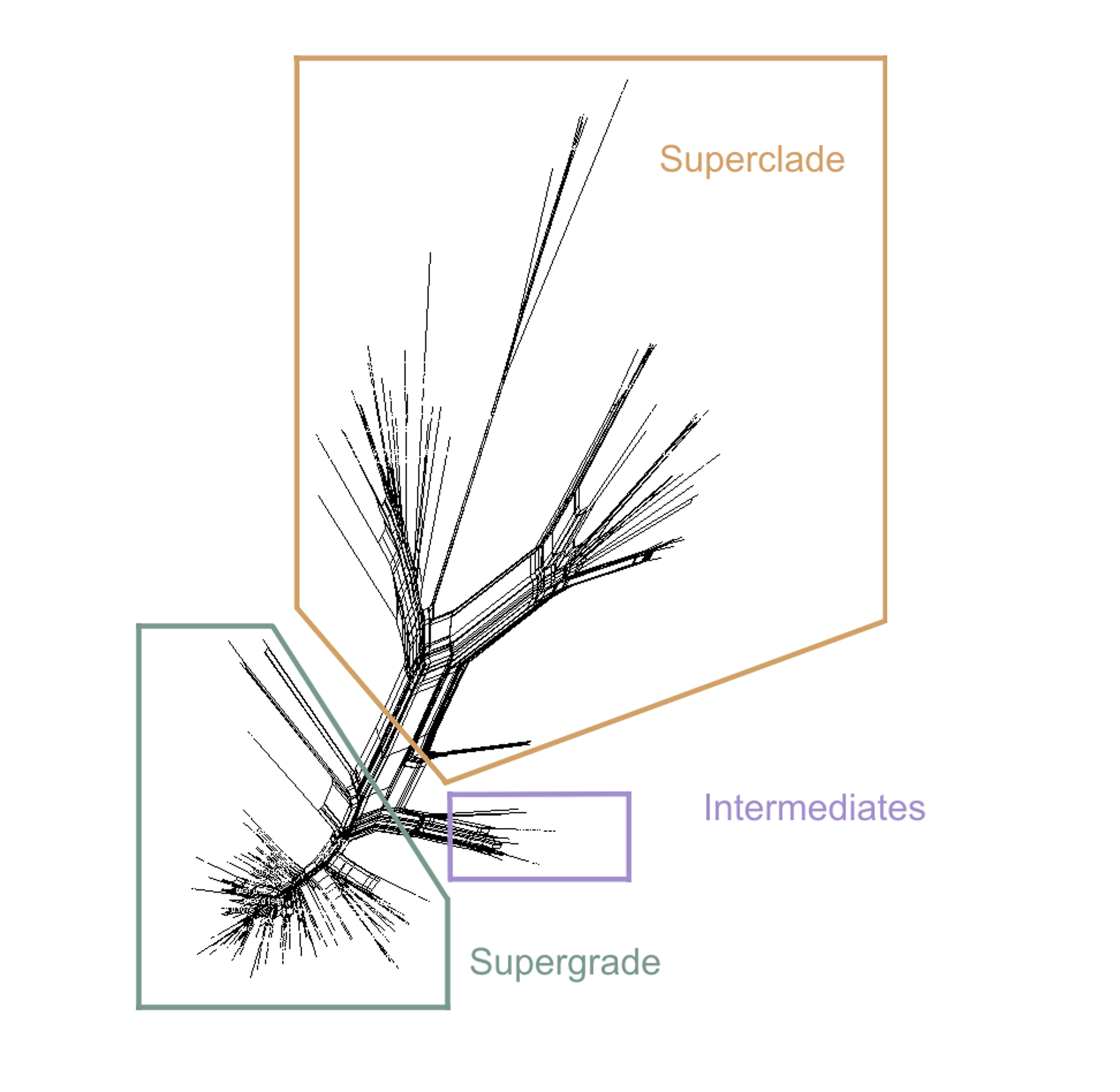

Supplement: Figure S2 — Splitstree reticulogram. The three major parts of the phylogeny (compare phylogenetic tree in Figs. 3, 4, 5) are labeled here. (TIF) [file pone.0088141.s002.tif]

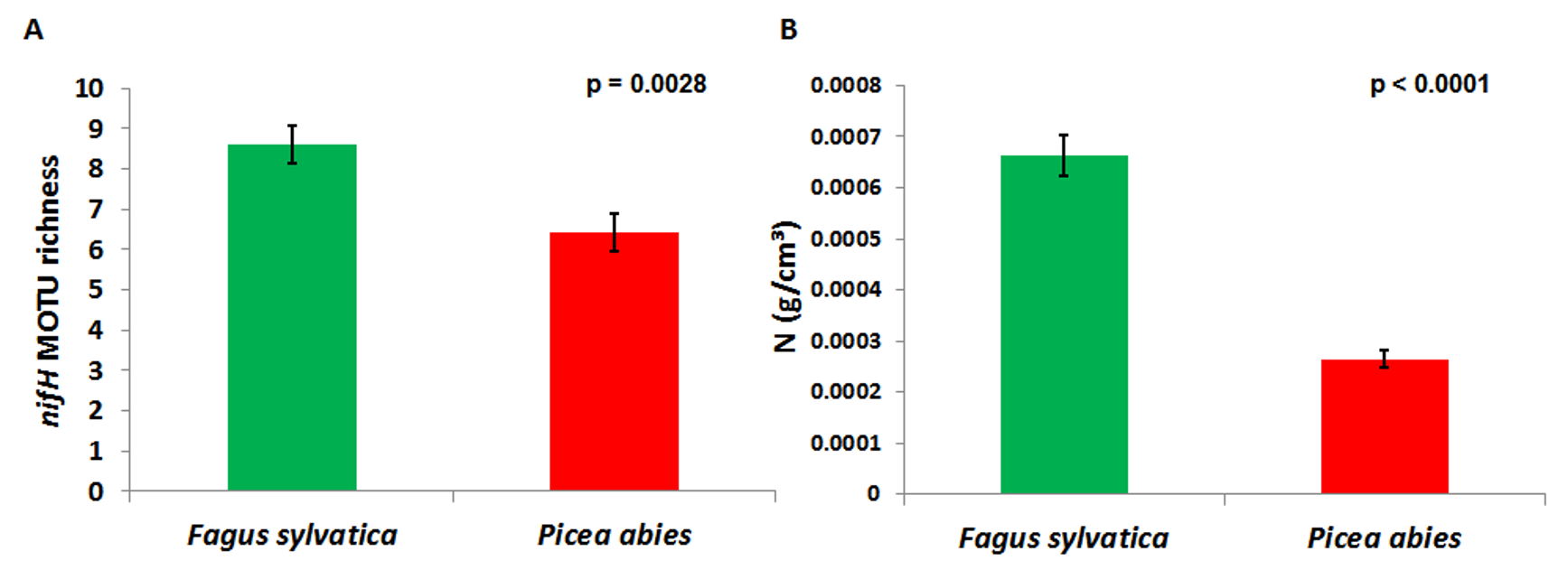

Supplement: Figure S3 — Bargraphs including standard errors displaying nifH MOTU richness (A) and nitrogen content per density unit (B) within dead wood tree species. (TIF) [file pone.0088141.s003.tif]

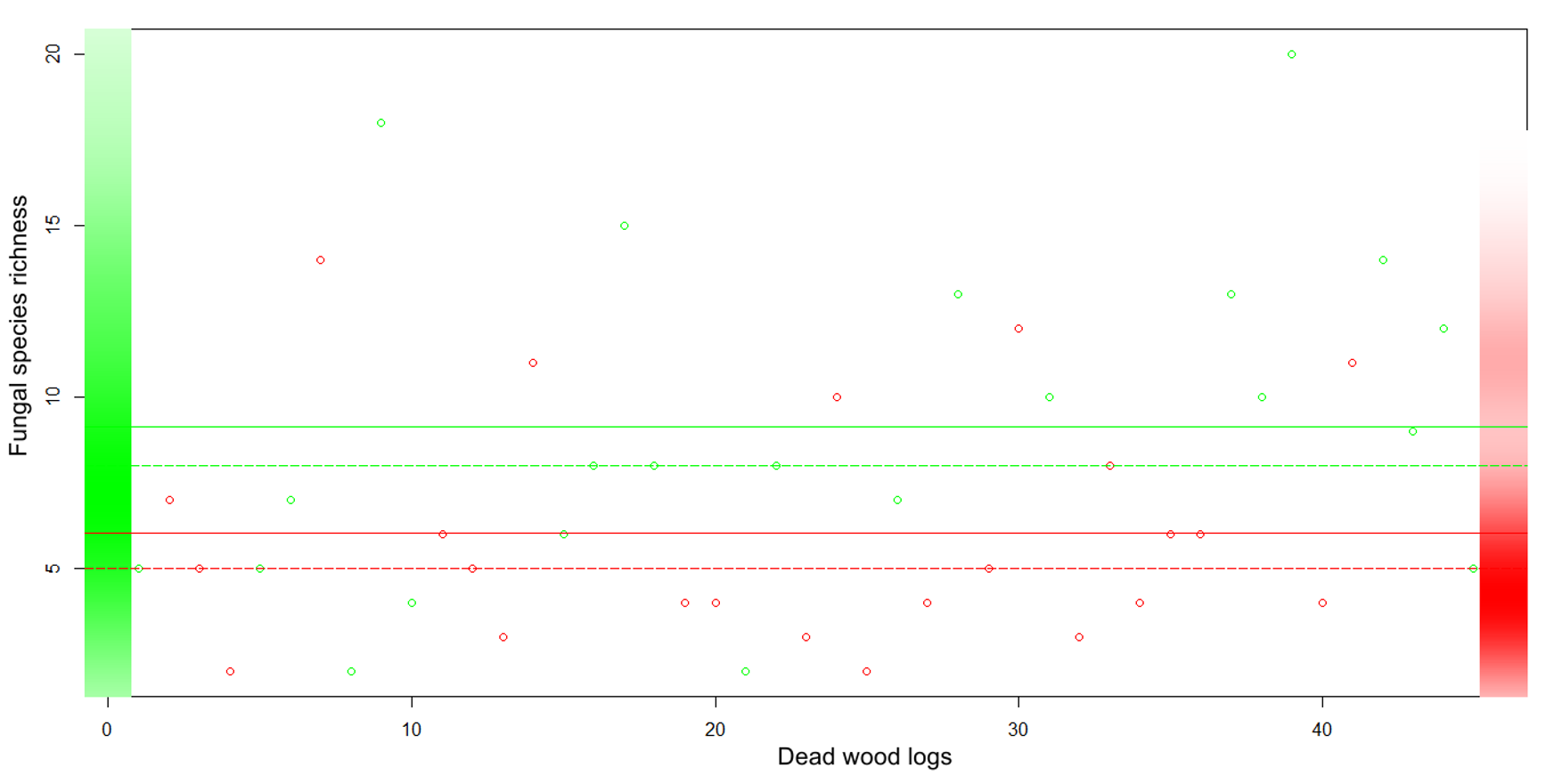

Supplement: Figure S4 — Scatterplot displaying number ( = richness) of fruiting fungal species per dead wood log. Solid vertical lines display mean values of richness, dashed vertical lines median richness per tree species (green = Fagus sylvatica, red = Picea abies). Heatmapped bars to the left and right display density probability as calculated by kernel density estimation using the denstrip package in R (Jackson CH (2008) Displaying uncertainty with shading. Am Stat 62: 340-347.). (TIF) [file pone.0088141.s004.tif]

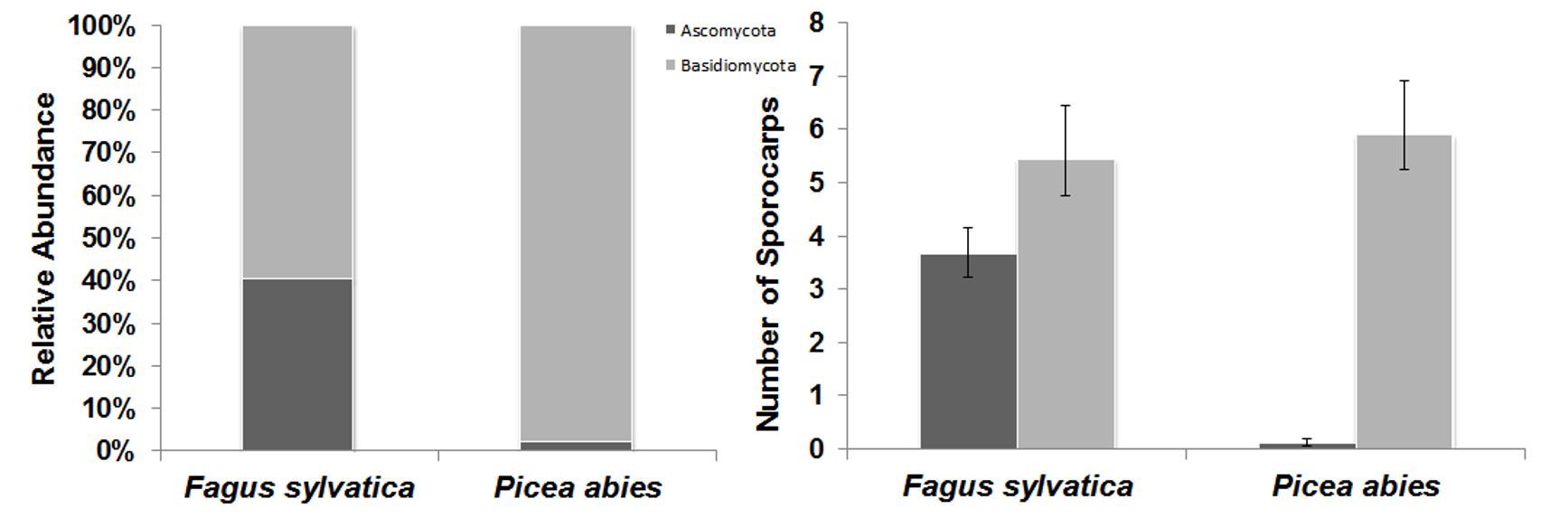

Supplement: Figure S5 — Relative abundances (left) of Basidiomycota and Ascomycota on dead wood logs of Fagus sylvatica and Picea abies and mean number of sporocarps per tree species (right). (TIF) [file pone.0088141.s005.tif]

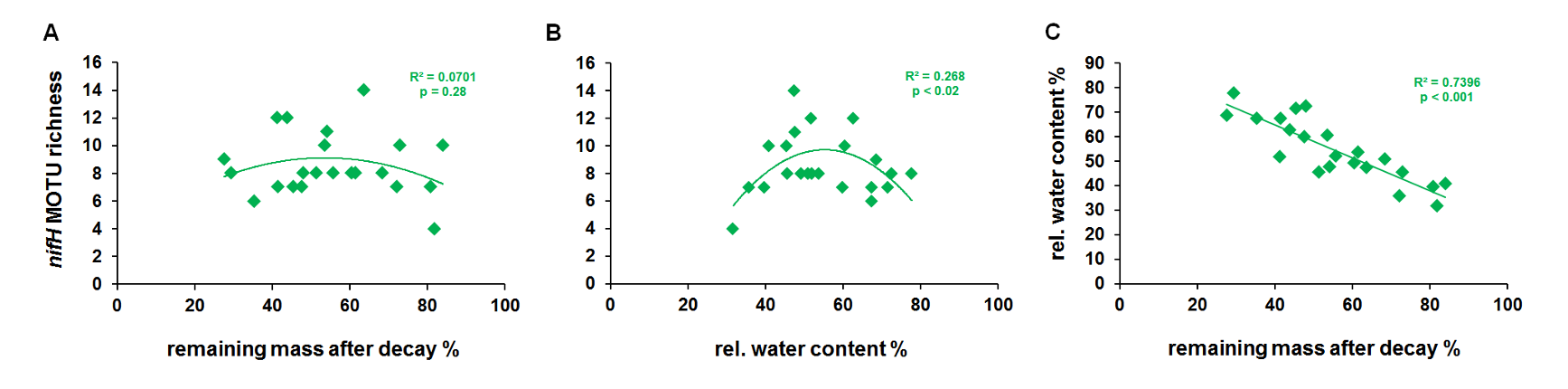

Supplement: Figure S6 — Interrelation of nifH MOTU richness and remaining mass after decay in % (A) and water content in % (B) and water content in % and remaining mass after decay in % (C) on logs of Fagus sylvatica . (TIF) [file pone.0088141.s006.tif]

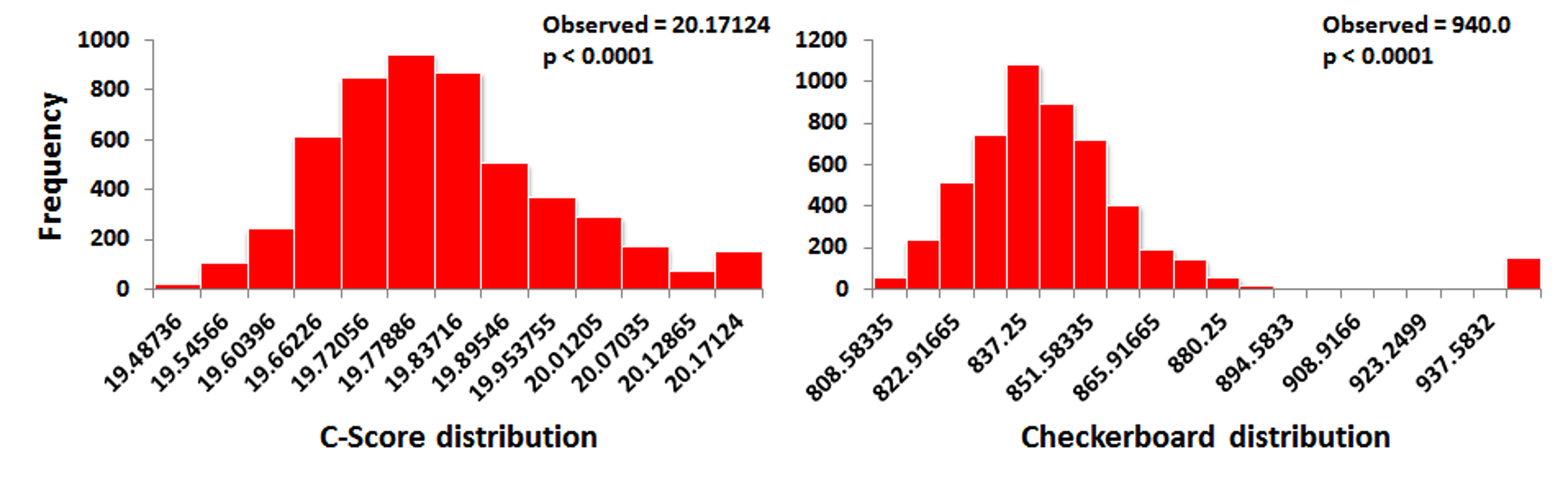

Supplement: Figure S7 — Non-random sporocarp – nifH MOTU community assembly assessed by C-score distribution and Checkerboard index for observed and expected/ randomized species occurrences. (TIF) [file pone.0088141.s007.tif]

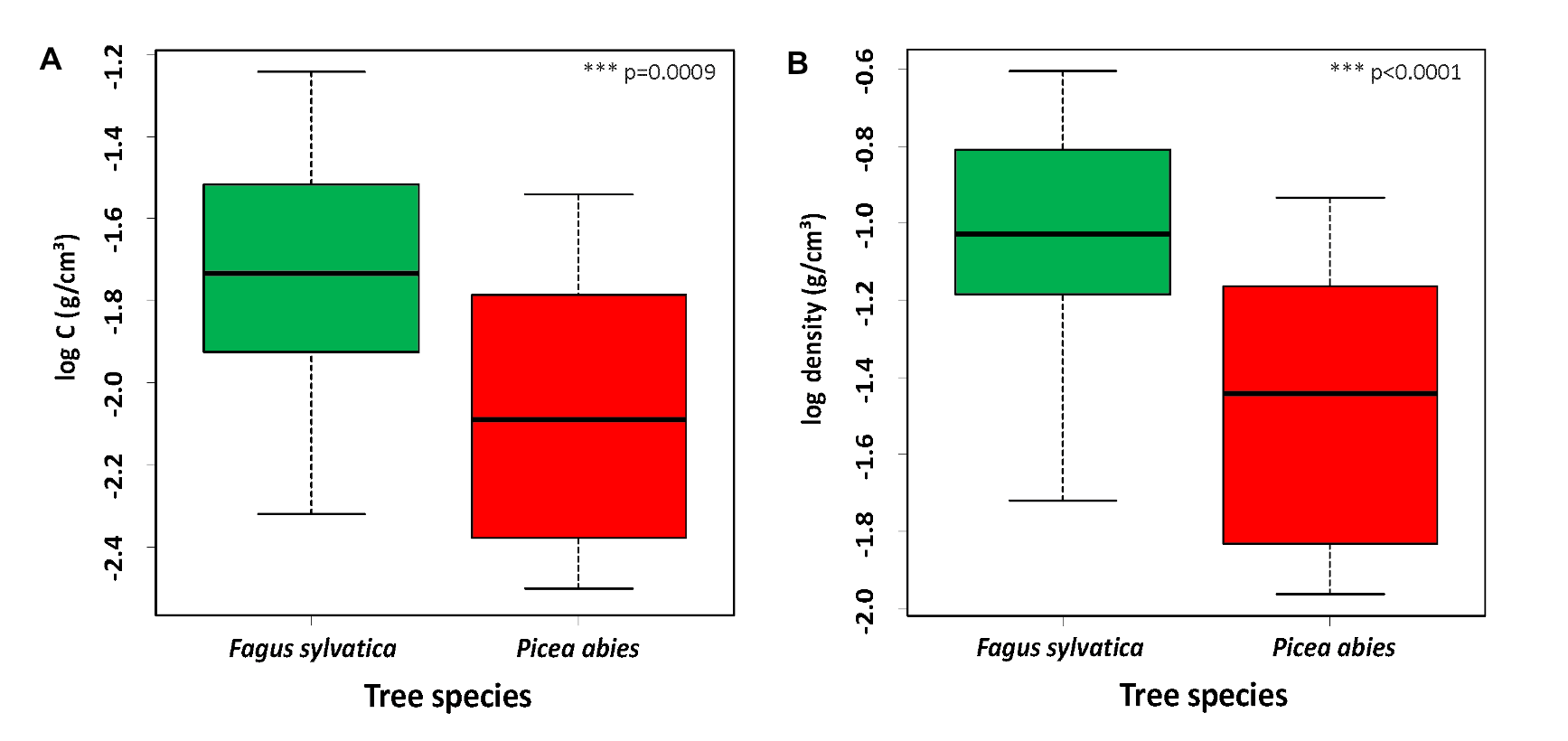

Supplement: Figure S8 — Boxplots including median, upper and under quartiles and whiskers displaying the interrelation of dead wood species and log transformed carbon content per density unit (A) and log transformed wood density (B). (TIF) [file pone.0088141.s008.tif]

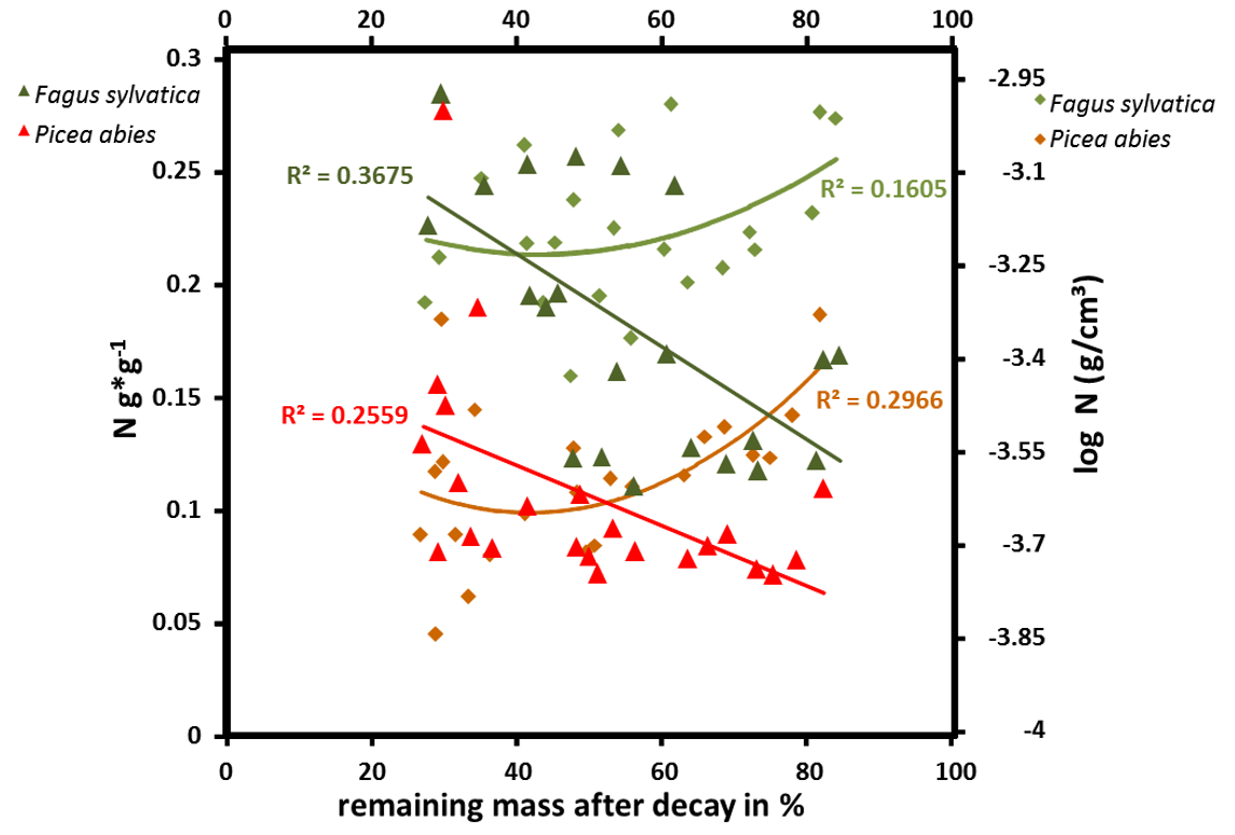

Supplement: Figure S9 — Effects of remaining mass after decay in % on log-transformed nitrogen content per density unit (g*cm3) and N concentration in g*g-1. Interrelations are displayed separately per wood species. (TIF) [file pone.0088141.s009.tif]
