# Supplementary material for: Network Analysis Reveals Ecological Links between N-Fixing Bacteria and Wood-Decaying Fungi
Source: PLoS One. 2014 Feb 5;9(2):e88141. doi: 10.1371/journal.pone.0088141 (PMC3914916; doi:10.1371/journal.pone.0088141)
Supplement: Table S1 — Sequence percentage identity of MOTUs taxonomically assigned through BLASTn against GenBank (uncultered/ environmental sample sequences excluded). 19 MOTUs were assigned to Rhizobiales at a 97% similarity threshold (65 MOTUs at ≥90%). A total of 80 MOTUs were identified to genus level. (DOCX) [file pone.0088141.s010.docx]

Tab. S1. Sequence percentage identity of MOTUs taxonomically assigned through blastn against GenBank (uncultered/ environmental sample sequences excluded). 19 MOTUs were assigned to Rhizobiales at a 97% similarity threshold (65 MOTUs at ≥90%). A total of 80 MOTUs were identified to genus level.

| **Order** | **Genus** | **100%** | **99%** | **98%** | **97%** | **96%** | **95%** | **90-94%** |
| --- | --- | --- | --- | --- | --- | --- | --- | --- |
| **Rhizobiales** | Bradyrhizobium | **1** |  |  | **1** | **2** | **4** | **28** |
|  | Methylocella |  | **1** |  |  |  |  |  |
|  | Methyloferula |  |  | **3** |  |  | **1** | **1** |
|  | Beijerinckiaceae* |  |  |  | **1** | **1** |  |  |
|  | Methylocapsa |  |  |  |  | **2** | **1** | **1** |
|  | Rhodomicrobium |  |  |  |  |  | **1** |  |
|  | Methylococcus |  |  |  |  |  |  | **1** |
|  | Methylobacterium |  |  |  |  |  |  | **2** |
|  | Methylocystis |  |  |  |  |  |  | **9** |
|  | Xanthobacter |  |  |  |  |  |  | **4** |
| **Rhodocyclales** | Azospira |  |  | **1** |  |  |  |  |
| **Pseudomonadales** | Pseudomonas |  |  |  |  | **1** |  |  |
| **Rhodospirillales** | Telmatospirillum |  |  |  |  | **1** | **2** | **2** |
|  | Azospirillum |  | **1** |  |  |  |  | **1** |
| **Sphingomonadales** | Sphingomonas |  |  |  |  |  |  | **2** |
| **Burkholderiales** | Burkholderia |  | **1** |  |  |  |  | **1** |
|  | Ideonella |  |  |  |  |  |  | **1** |
|  | Pelomonas |  |  |  |  |  |  | **1** |

*no subfamilial resolution
