# Supplementary material for: Network Analysis Reveals Ecological Links between N-Fixing Bacteria and Wood-Decaying Fungi
Source: PLoS One. 2014 Feb 5;9(2):e88141. doi: 10.1371/journal.pone.0088141 (PMC3914916; doi:10.1371/journal.pone.0088141)
Supplement: Table S2 — List of sporocarps identified on the respective dead wood trees A) Fagus sylvatica B) Picea abies. (DOCX) [file pone.0088141.s011.docx]

Tab. S2: List of sporocarps identified on the respective dead wood trees A) *Fagus sylvatica* B) *Picea abies*.

**A) *Fagus sylvatica***

| **CWD_ID *Fagus sylvatica*** | **08270** | **08277** | **08280** | **08283** | **08285** | **08286** | **08295** | **08296** | **08297** | **08298** | **08807** | **08810** | **08815** | **08818** | **08821** | **08827** | **08828** | **08832** | **08835** | **08842** | **08845** | **08846** |
| --- | --- | --- | --- | --- | --- | --- | --- | --- | --- | --- | --- | --- | --- | --- | --- | --- | --- | --- | --- | --- | --- | --- |
| ***Antrodiella hoehnelii*** |  |  |  |  |  |  |  |  |  |  |  |  |  |  |  | X |  |  |  |  |  |  |
| ***Armillaria spec.*** | X |  | X | X |  |  |  |  |  |  |  | X | X | X | X | X | X |  |  | X | X | X |
| ***Ascocoryne cylichnium*** |  |  |  |  |  |  |  |  |  |  |  |  |  |  |  | X |  |  |  |  |  |  |
| ***Ascocoryne spec.*** |  |  | X |  |  |  |  | X | X |  |  | X | X | X |  |  |  | X |  | X | X | X |
| ***Ascodichaena rugosa*** |  |  |  |  | X |  |  |  |  |  |  |  |  |  |  |  |  |  |  |  |  |  |
| ***Athelia epiphylla*** |  |  |  |  |  |  | X | X |  |  |  |  |  |  |  |  |  |  |  |  |  |  |
| ***Bispora monilioides*** |  |  |  |  |  |  |  |  |  |  |  | X |  | X |  |  |  | X |  |  | X |  |
| ***Bisporella citrina*** |  |  |  |  |  |  |  |  | X |  |  |  | X |  |  | X |  |  |  |  |  |  |
| ***Bjerkandera adusta*** |  |  |  |  |  |  |  |  |  |  |  | X |  |  |  |  |  | X |  |  |  |  |
| ***Bjerkandera spec.*** |  |  |  |  |  |  |  |  |  |  |  |  |  |  |  |  |  | X |  |  |  |  |
| ***Botryobasidium aureum*** |  |  |  |  |  |  |  |  |  |  |  |  |  |  |  |  |  |  |  |  | X |  |
| ***Botryobasidium vagum*** |  |  |  |  |  |  |  |  | X |  |  |  |  |  |  |  |  |  |  |  |  |  |
| ***Calocera cornea*** |  |  |  |  |  |  |  |  |  |  |  |  |  |  |  |  | X |  |  |  |  |  |
| ***Ceriporia excelsa*** |  |  |  |  |  |  |  |  |  |  |  |  |  |  |  |  |  |  | X |  |  |  |
| ***Ceriporia purpurea*** |  |  |  |  | X |  |  |  |  |  |  |  |  |  |  |  |  |  |  |  |  |  |
| ***Ceriporia spec.*** |  |  |  |  | X |  |  |  | X |  |  |  |  |  |  |  |  |  |  |  |  |  |
| ***Ceriporiopsis spec.*** |  |  |  |  | X |  |  |  |  |  |  |  |  |  |  |  |  |  |  |  |  |  |
| ***Coprinellus micaceus*** |  |  |  |  |  |  |  |  |  |  |  |  |  |  |  |  |  | X |  |  |  |  |
| ***Coprinus spec.*** |  |  |  |  |  |  |  |  |  |  |  |  |  | X |  |  |  |  |  |  |  |  |
| ***Dacrymyces spec.*** |  |  |  |  |  |  | X |  |  |  |  |  |  |  |  |  |  |  |  |  |  |  |
| ***Datronia mollis*** |  |  |  |  |  |  |  |  |  |  |  |  |  |  |  |  | X |  |  |  |  |  |
| ***Eutypella quaternata*** |  |  |  |  |  |  |  |  |  |  |  |  |  |  |  |  |  | X |  |  |  |  |
| ***Exidia glandulosa*** |  |  |  |  |  |  |  |  |  |  |  |  |  |  | X |  |  |  |  |  |  |  |
| ***Fomes fomentarius*** |  |  | X |  | X |  |  |  | X |  |  |  |  |  |  |  |  | X |  |  |  |  |
| ***Fomitopsis pinicola*** |  |  |  |  | X |  |  |  | X |  |  |  | X |  |  |  |  |  |  |  |  |  |
| ***Galerina spec.*** |  |  |  |  | X |  |  |  |  |  |  |  |  |  |  |  |  |  | X |  |  |  |
| ***Ganoderma applanatum*** |  |  |  |  | X |  |  |  | X |  |  |  |  |  |  |  |  | X |  |  |  |  |
| ***Grandinia spec.*** | X |  |  |  |  |  |  |  |  |  |  |  |  |  |  |  |  |  |  |  |  |  |
| ***Hyalorbilia inflatula*** |  |  |  |  |  |  |  |  |  |  |  |  |  |  |  |  |  |  |  | X |  |  |
| ***Hymenoscyphus spec.*** |  |  |  |  |  |  |  |  |  |  |  |  |  | X |  |  |  |  |  |  |  |  |
| ***Hyphodontia sambuci*** |  |  |  |  |  |  |  |  |  |  |  |  |  |  |  | X |  |  |  |  |  |  |
| ***Hypholoma fasciculare*** |  |  |  |  | X |  |  |  |  |  |  |  |  |  |  |  |  |  |  |  |  |  |
| ***Hypholoma lateritium*** |  |  |  |  |  |  |  |  |  |  |  |  |  |  |  |  |  | X |  |  |  |  |
| ***Hypochnicium polonense*** |  |  |  |  |  |  |  |  |  |  |  |  |  |  |  |  |  |  | X |  |  |  |
| ***Hypocrea minutispora*** |  |  |  |  |  |  |  |  |  | X |  |  |  |  | X |  |  |  |  |  |  |  |
| ***Hypocrea spec.*** |  |  |  |  |  |  |  |  |  |  |  | X |  |  |  |  |  |  |  |  |  |  |
| ***Hypoxylon cohaerens*** |  |  |  |  |  |  |  |  | X |  | X |  |  |  |  | X | X | X |  |  |  |  |
| ***Hypoxylon fragiforme*** |  | X |  |  |  |  |  |  |  |  |  |  |  |  | X |  |  |  |  | X | X |  |
| ***Hypoxylon rubiginosum*** |  |  |  |  |  |  |  | X |  | X |  |  |  |  | X | X |  |  |  |  |  |  |
| ***Hypoxylon spec.*** |  |  | X |  |  |  |  |  |  |  |  |  |  |  | X |  |  |  |  |  |  |  |
| ***Kretzschmaria deusta*** |  |  |  |  | X | X |  |  | X | X |  |  |  |  |  |  |  | X | X |  |  |  |
| ***Kuehneromyces mutabilis*** |  |  |  |  |  |  |  |  |  |  |  |  |  | X |  |  |  |  | X |  |  |  |
| ***Lenzites betulina*** |  |  |  |  |  |  |  |  |  |  |  |  |  |  |  |  | X |  |  |  |  |  |
| ***Lycoperdon pyriforme*** |  |  |  |  |  |  |  |  |  |  |  |  |  |  |  |  |  |  | X |  |  |  |
| ***Marasmiellus spec.*** |  |  |  |  |  |  |  |  |  |  |  |  |  |  |  |  |  |  | X |  |  |  |
| ***Marasmius alliaceus*** |  |  | X |  | X | X |  |  | X | X |  |  |  |  |  |  |  | X |  |  |  |  |
| ***Megacollybia platyphylla*** |  |  |  |  |  | X |  |  |  | X |  |  |  |  |  |  |  |  |  |  | X |  |
| ***Melogramma spiniferum*** |  |  |  |  |  |  |  |  |  |  |  |  |  |  |  |  |  |  | X |  |  |  |
| ***Mollisia ligni*** | X |  |  |  | X |  |  |  |  |  |  |  |  |  |  |  |  |  |  |  |  |  |
| ***Mollisia spec.*** |  |  |  |  |  |  |  |  |  |  |  |  |  |  |  |  |  |  |  |  | X |  |
| ***Mutatoderma mutatum*** |  |  |  |  |  |  |  |  |  |  |  |  |  |  |  | X |  |  |  |  |  |  |
| ***Mycena galericulata*** |  |  |  |  |  |  |  |  |  |  |  |  |  | X |  |  |  |  |  | X |  |  |
| ***Mycena speirea*** |  |  |  |  |  |  |  | X |  |  |  |  |  | X |  |  |  |  |  |  |  |  |
| ***Mycena crocata*** |  |  |  |  |  |  |  |  | X |  |  |  |  |  |  |  |  |  | X |  |  |  |
| ***Mycena renati*** |  |  |  |  |  |  |  | X |  |  |  |  |  |  |  |  |  |  | X |  |  |  |
| ***Mycena rubromarginata*** |  |  |  |  |  |  |  |  |  |  |  | X |  |  |  |  |  |  |  |  |  |  |
| ***Mycena spec.*** |  |  |  |  |  |  |  |  |  |  |  |  |  |  |  |  |  |  |  |  |  | X |
| ***Nectria spec.*** |  |  |  |  |  |  | X |  |  |  |  |  |  |  |  |  |  |  |  |  |  |  |
| ***Nemania confluens*** |  |  |  |  |  |  |  |  |  |  |  |  |  |  |  | X |  |  |  |  |  |  |
| ***Nemania serpens*** |  |  |  |  | X |  |  | X |  | X |  |  | X |  |  | X |  | X |  |  |  |  |
| ***Neobulgaria pseudoombrophila*** |  |  |  |  |  |  |  |  |  |  |  |  |  |  | X |  |  |  |  |  |  |  |
| ***Neobulgaria pura*** |  | X |  |  |  |  |  |  |  |  |  |  | X |  |  |  |  |  |  |  |  |  |
| ***Neodasyscypha cerina*** |  |  |  |  |  |  |  |  |  |  |  |  |  |  | X |  | X |  |  |  |  |  |
| ***Orbilia spec.*** |  |  |  |  |  |  |  |  |  |  |  |  |  |  |  |  |  | X |  | X | X | X |
| ***Oudemansiella mucida*** |  |  |  |  |  |  |  |  |  |  |  |  |  |  |  | X |  |  |  |  |  |  |
| ***Panellus serotinus*** |  |  | X |  |  |  |  |  |  |  |  |  |  | X |  |  |  | X |  |  |  |  |
| ***Peniophorella guttulifera*** |  |  |  |  |  |  |  |  |  |  |  |  |  |  |  |  |  |  | X |  |  |  |
| ***Peziza obtusapiculata*** |  |  |  |  |  |  |  |  |  |  |  |  |  |  |  |  |  | X |  |  |  |  |
| ***Phanerochaete velutina*** |  |  |  |  | X |  |  |  |  |  |  |  |  |  |  |  |  |  |  |  |  |  |
| ***Phellinus ferruginosus*** |  |  |  |  |  |  |  |  |  |  |  |  |  |  |  |  |  |  |  |  | X |  |
| ***Phlebia livida*** |  |  |  |  |  |  | X |  |  |  |  |  |  |  |  |  |  |  |  |  |  |  |
| ***Physisporinus vitreus*** |  |  |  |  | X |  |  |  |  |  |  |  |  |  |  |  |  |  |  |  |  |  |
| ***Pluteus pouzerianus*** | X |  |  |  |  |  |  |  |  |  |  |  |  |  |  |  |  |  |  |  |  |  |
| ***Pluteus phlebophorus*** |  |  |  |  |  |  | X |  | X |  |  |  |  |  |  |  |  |  |  |  |  |  |
| ***Pluteus salicinus*** |  |  |  |  |  |  |  |  |  |  |  |  |  |  |  |  |  |  |  | X |  |  |
| ***Pluteus spec.*** |  |  |  |  |  |  |  |  |  |  |  |  |  |  |  |  |  | X |  |  |  |  |
| ***Postia subcaesia*** |  |  |  |  |  |  |  |  |  |  |  |  |  |  |  |  | X |  |  |  |  |  |
| ***Psathyrella rostellata*** |  |  |  |  |  | X |  |  |  |  |  |  |  |  |  |  |  |  | X |  |  |  |
| ***Pycnoporus cinnabarinus*** |  |  |  |  |  |  |  |  |  |  |  |  |  |  |  |  | X |  |  |  |  |  |
| ***Ruzenia spermoides*** |  |  |  |  |  |  |  |  |  |  |  |  |  |  |  |  |  |  |  |  | X |  |
| ***Schizopora paradoxa*** |  | X |  |  |  |  |  |  |  |  |  |  |  |  |  |  |  |  |  |  |  |  |
| ***Scopuloides hydnoides*** |  |  |  |  |  |  |  |  |  | X |  |  |  |  | X |  |  |  |  |  | X | X |
| ***Scutellinia scutellata*** |  |  |  |  | X |  |  |  |  |  |  |  |  |  |  |  |  |  |  |  |  |  |
| ***Sebacina incrustans*** |  |  |  |  |  |  |  |  |  |  |  |  | X |  |  |  |  |  |  |  |  |  |
| ***Sistotrema brinkmannii*** |  |  |  |  |  |  |  | X |  |  | X |  |  |  |  |  |  |  |  |  |  |  |
| ***Stereum rugosum*** |  |  |  |  |  |  |  |  |  |  |  |  |  |  |  |  |  |  | X |  |  |  |
| ***Stereum spec.*** |  |  |  |  |  |  |  |  |  |  |  |  |  |  |  |  |  |  |  |  | X |  |
| ***Stereum subtomentosum*** |  |  |  |  |  |  |  |  |  |  |  |  |  |  |  | X |  |  |  |  |  |  |
| ***Stypella grilletii*** |  |  |  |  |  |  | X |  |  |  |  |  |  |  |  |  |  |  |  |  |  |  |
| ***Subulicystidium longisporum*** |  |  |  |  |  |  |  |  |  |  |  | X |  |  |  |  |  |  |  |  |  |  |
| ***Tomentella bryophila*** |  |  |  | X |  |  |  |  |  |  |  |  |  |  |  |  |  |  |  |  |  |  |
| ***Trametes gibbosa*** |  |  |  |  | X |  |  |  |  |  |  |  |  | X |  |  |  | X |  |  |  |  |
| ***Trametes hirsuta*** |  |  |  |  |  |  |  |  |  | X |  |  |  | X |  |  | X |  |  |  |  |  |
| ***Trametes versicolor*** |  | X |  |  |  |  |  |  | X |  |  |  |  | X |  |  | X | X |  |  |  |  |
| ***Trechispora nivea*** |  |  |  |  |  |  |  |  | X |  |  |  |  |  |  |  |  |  |  |  |  |  |
| ***Tubaria furfuracea*** |  |  |  |  |  |  |  |  |  |  |  |  |  |  |  | X |  |  |  |  |  |  |
| ***Xenasmatella vaga*** |  |  |  |  |  |  |  | X |  |  |  |  |  |  |  |  |  |  |  | X |  |  |
| ***Xylaria hypoxylon*** | X | X | X |  | X |  |  |  | X |  |  | X |  | X | X |  |  | X | X | X |  |  |

**B) *Picea abies***

| **CWD_ID *Picea abies*** | **08271** | **08273** | **08276** | **08281** | **08288** | **08289** | **08290** | **08294** | **08801** | **08804** | **08811** | **08812** | **08813** | **08817** | **08819** | **08820** | **08822** | **08823** | **08824** | **08825** | **08826** | **08833** | **08834** |
| --- | --- | --- | --- | --- | --- | --- | --- | --- | --- | --- | --- | --- | --- | --- | --- | --- | --- | --- | --- | --- | --- | --- | --- |
| ***Amylostereum areolatum*** |  |  |  |  |  |  |  |  |  |  |  |  | X |  |  |  |  | X | X |  |  |  |  |
| ***Amylostereum spec.*** |  |  |  |  |  |  |  |  |  | X |  |  |  |  |  |  |  |  |  |  |  |  |  |
| ***Armillaria spec.*** |  |  |  | X |  |  |  |  |  |  |  | X | X | X |  |  |  |  |  |  | X |  | X |
| ***Athelia decipiens*** |  |  |  |  | X |  |  |  |  |  |  |  |  |  |  |  |  |  |  |  |  |  |  |
| ***Athelia epiphylla*** |  |  |  |  |  |  |  |  |  |  |  |  |  |  |  |  |  |  |  |  |  | X |  |
| ***Boidinia furfuracea*** |  |  |  |  |  |  |  | X |  |  |  |  |  |  |  |  |  |  |  |  |  |  |  |
| ***Botryobasidium subcoronatum*** | X |  |  | X |  | X |  | X |  |  |  |  |  |  |  |  |  |  |  |  |  |  |  |
| ***Botryobasidium vagum*** |  |  |  |  |  |  |  |  | X |  | X |  |  |  | X |  |  |  |  |  | X |  | X |
| ***Botryobasidium laeve*** |  |  |  |  |  |  |  |  | X |  |  |  |  |  |  |  |  |  |  |  |  |  |  |
| ***Cabalodontia subcretacea*** |  |  |  |  |  |  |  |  |  |  |  |  |  |  |  |  |  |  |  |  |  |  | X |
| ***Camarops tubulina*** |  |  |  |  |  |  |  |  |  |  |  |  |  |  |  |  |  |  |  |  |  |  | X |
| ***Ceriporiopsis mucida*** |  |  |  |  |  |  |  |  |  |  |  | X |  |  |  |  |  |  |  |  |  |  |  |
| ***Chrysomphalina grossula*** |  |  |  |  |  |  |  |  |  |  |  |  |  |  |  |  | X |  |  |  |  |  |  |
| ***Clavulicium delectabile*** |  |  |  |  |  |  |  |  |  |  |  |  |  |  |  |  |  | X |  |  |  |  |  |
| ***Dacrymyces stillatus*** |  | X |  |  |  | X |  |  |  |  |  |  |  |  |  |  |  |  | X |  | X |  |  |
| ***Dacryobolus sudans*** |  | X |  |  |  |  |  |  |  |  |  |  |  |  |  |  |  |  |  |  |  |  |  |
| ***Exidiopsis spec.*** |  |  |  |  |  |  |  |  |  |  |  |  |  |  |  |  |  |  |  |  |  |  | X |
| ***Fomitopsis pinicola*** |  |  |  | X | X |  | X | X |  |  |  | X |  |  |  |  |  |  |  |  |  |  | X |
| ***Galerina marginata*** |  |  |  |  |  |  |  |  | X |  |  |  |  |  |  |  |  |  |  |  |  |  |  |
| ***Galerina spec.*** |  |  |  |  |  |  |  |  |  |  |  |  |  |  |  |  |  |  |  |  |  | X |  |
| ***Ganoderma applanatum*** |  |  |  |  |  |  |  |  |  |  |  |  |  |  |  |  |  |  |  |  |  |  | X |
| ***Gloeophyllum sepiarium*** |  |  |  |  |  |  |  |  |  |  |  |  |  |  |  |  |  |  |  | X |  |  |  |
| ***Gymnopilus penetrans*** |  |  |  | X |  |  | X |  |  |  |  |  |  |  |  |  |  |  |  |  |  |  |  |
| ***Henningsomyces candidus*** |  |  |  |  |  |  |  |  |  |  |  |  |  |  |  | X |  |  |  |  |  |  |  |
| ***Heterobasidion annosum*** |  |  |  |  | X |  |  | X |  |  |  |  |  |  |  | X | X |  |  |  | X |  |  |
| ***Hyphoderma argillaceum*** |  |  |  |  |  | X |  |  |  |  |  |  |  |  |  | X |  | X |  |  |  |  |  |
| ***Hyphodontia alutaria*** |  |  |  |  |  |  |  |  |  |  |  |  |  |  |  |  |  | X |  |  |  |  |  |
| ***Hyphodontia breviseta*** |  |  |  |  |  |  |  |  |  |  |  |  |  |  |  | X |  |  |  |  |  |  |  |
| ***Hyphodontia nespori*** | X |  |  |  |  |  |  |  |  |  |  |  |  |  |  |  |  |  |  |  |  |  | X |
| ***Hyphodontia pallidula*** |  | X |  |  |  |  |  |  |  |  |  |  |  |  |  |  |  |  |  |  |  |  |  |
| ***Hyphodontia spathulata*** |  |  |  |  |  |  |  |  |  |  |  |  |  |  |  | X |  |  |  |  |  |  |  |
| ***Hyphodontia spec.*** |  |  |  |  |  |  |  |  |  |  |  |  |  |  |  |  |  |  |  |  |  |  | X |
| ***Hypholoma capnoides*** | X |  |  |  |  |  |  |  |  |  |  |  |  | X |  | X |  |  |  |  |  |  |  |
| ***Hypocrea pulvinata*** |  |  |  |  |  |  |  | X |  |  |  |  |  |  |  |  |  |  |  |  |  |  |  |
| ***Ischnoderma benzoinum*** |  |  |  |  |  |  |  |  |  |  |  |  |  |  |  |  |  |  |  |  |  | X |  |
| ***Marasmius androsaceus*** |  |  |  |  |  |  |  |  |  |  |  |  |  |  |  |  |  |  | X |  |  |  |  |
| ***Mucronella spec.*** |  |  |  |  |  |  |  |  |  |  |  |  |  |  | X |  |  |  |  |  |  |  |  |
| ***Mycena stipata*** |  |  |  |  |  |  |  |  |  |  |  |  |  |  |  | X |  |  |  |  |  |  |  |
| ***Mycena cyanorhiza*** |  |  |  |  |  |  |  |  |  |  |  |  |  |  |  | X |  |  |  |  |  |  |  |
| ***Mycena metata*** |  | X |  | X |  |  |  |  |  | X |  |  |  |  |  | X |  | X |  |  |  |  | X |
| ***Mycena rubromarginata*** | X |  | X |  |  |  |  |  |  |  |  |  |  |  |  |  |  |  |  |  |  |  |  |
| ***Mycena spec.*** |  |  |  |  |  |  |  |  |  |  | X |  |  | X |  |  |  |  |  |  |  |  |  |
| ***Neobulgaria spec.*** |  |  |  |  |  |  |  |  |  |  |  | X |  |  |  |  |  |  |  |  |  |  |  |
| ***Peniophorella pallida*** |  |  |  |  |  |  |  |  |  |  |  | X |  |  |  |  |  |  |  |  |  |  | X |
| ***Peniophorella praetermissa*** |  |  |  | X |  |  |  |  |  |  |  |  |  |  |  |  |  |  |  |  | X |  |  |
| ***Pholiota flammans*** |  |  |  |  |  |  |  |  |  |  |  |  |  |  |  |  |  |  |  |  |  | X |  |
| ***Physisporinus spec.*** |  |  |  |  |  |  |  |  |  |  |  |  |  |  | X |  |  |  |  |  |  |  |  |
| ***Piloderma bicolor*** |  |  |  |  |  |  |  | X |  |  |  |  |  |  |  |  |  |  |  |  |  |  |  |
| ***Postia caesia*** | X | X | X |  |  |  |  |  |  |  |  |  |  | X |  |  |  | X |  |  |  |  |  |
| ***Postia stiptica*** |  |  |  | X |  |  |  |  |  |  |  | X |  |  |  |  |  |  |  |  |  |  |  |
| ***Postia tephroleuca*** |  |  |  |  |  |  |  |  |  |  |  |  |  |  |  |  |  |  |  | X |  |  |  |
| ***Pycnoporellus fulgens*** |  |  |  |  | X |  |  | X |  |  |  |  |  |  |  |  |  |  |  |  |  |  |  |
| ***Resinicium bicolor*** | X |  |  | X |  | X |  | X |  | X | X | X |  |  |  | X |  | X |  | X | X |  |  |
| ***Skeletocutis carneogrisea*** |  |  |  | X |  |  |  |  |  |  |  |  |  |  |  |  |  |  |  |  |  |  |  |
| ***Skeletocutis kuehneri*** |  |  |  |  |  |  |  |  |  |  |  |  |  |  | X |  |  |  |  |  |  |  |  |
| ***Skeletocutis nivea*** |  |  |  |  |  |  |  |  |  |  |  | X |  |  |  |  |  |  |  |  |  |  |  |
| ***Skeletocutis spec.*** |  |  |  | X |  |  |  |  |  |  |  |  |  |  | X |  |  |  |  |  |  |  |  |
| ***Steccherinum ochraceum*** |  |  |  |  |  |  |  |  |  |  |  | X |  |  |  |  |  |  |  |  |  |  |  |
| ***Thanatephorus fusisporus*** |  |  |  |  |  |  |  |  |  |  |  | X |  |  |  |  |  |  |  |  |  |  |  |
| ***Tomentella lilacinogrisea*** |  |  |  |  |  |  |  | X |  |  |  |  |  |  |  |  |  |  |  |  |  |  |  |
| ***Tomentella spec.*** |  |  |  |  |  |  |  | X |  |  |  |  |  |  |  |  |  |  |  |  |  |  |  |
| ***Trechispora hymenocystis*** |  |  |  | X |  |  |  |  |  |  |  |  |  |  |  |  |  |  |  | X |  |  |  |
| ***Trechispora mollusca*** |  |  |  | X |  |  |  |  |  |  |  |  |  |  |  |  |  |  |  |  |  |  |  |
| ***Trechispora spec.*** |  |  |  |  |  |  |  |  |  |  |  |  |  |  |  | X |  |  |  |  |  |  |  |
| ***Trichaptum abietinum*** |  |  |  | X |  |  |  |  | X |  |  |  |  |  |  |  |  |  |  | X |  |  |  |
| ***Trichaptum fuscoviolaceum*** |  |  |  |  | X |  |  |  |  |  |  |  |  |  |  |  |  |  |  |  |  |  |  |
| ***Vesiculomyces citrinus*** |  |  |  |  |  |  | X |  |  | X |  |  |  |  |  |  |  |  |  |  |  |  |  |
| ***Xenasma tulasnelloidea*** |  |  |  |  |  | X |  |  |  |  |  |  |  |  |  | X |  |  |  |  |  |  |  |
| ***Xenasmatella vaga*** |  |  |  | X | X |  |  | X |  |  |  |  |  |  |  |  | X | X | X | X |  |  |  |
