# Supplementary material for: Correction: Network Analysis Reveals Ecological Links between N-Fixing Bacteria and Wood-Decaying Fungi
Source: PLoS One. 2014 Mar 14;9(3):e91389. doi: 10.1371/journal.pone.0091389 (PMC3954724; doi:10.1371/journal.pone.0091389)
Supplement: Table S1 — Sequence percentage identity of MOTUs taxonomically assigned through BLASTn against GenBank (uncultured/ environmental sample sequences excluded). 19 MOTUs were assigned to Rhizobiales at a 95% similarity threshold (65 MOTUs at ≥90%). A total of 80 MOTUs were identified to genus level. (DOCX) [file pone.0091389.s001.docx]

Tab. S1. Sequence percentage identity of MOTUs taxonomically assigned through BLASTn against GenBank (uncultured/ environmental sample sequences excluded). 19 MOTUs were assigned to Rhizobiales at a 95% similarity threshold (65 MOTUs at ≥90%). A total of 80 MOTUs were identified to genus level.

| **Order** | **Genus** | **100%** | **99%** | **98%** | **97%** | **96%** | **95%** | **90-94%** |
| --- | --- | --- | --- | --- | --- | --- | --- | --- |
| **Rhizobiales** | *Bradyrhizobium* | **1** |  |  | **1** | **2** | **4** | **28** |
|  | *Methylocella* |  | **1** |  |  |  |  |  |
|  | *Methyloferula* |  |  | **3** |  |  | **1** | **1** |
|  | Beijerinckiaceae* |  |  |  | **1** | **1** |  |  |
|  | *Methylocapsa* |  |  |  |  | **2** | **1** | **1** |
|  | *Rhodomicrobium* |  |  |  |  |  | **1** |  |
|  | *Methylococcus* |  |  |  |  |  |  | **1** |
|  | *Methylobacterium* |  |  |  |  |  |  | **2** |
|  | *Methylocystis* |  |  |  |  |  |  | **9** |
|  | *Xanthobacter* |  |  |  |  |  |  | **4** |
| **Rhodocyclales** | *Azospira* |  |  | **1** |  |  |  |  |
| **Pseudomonadales** | *Pseudomonas* |  |  |  |  | **1** |  |  |
| **Rhodospirillales** | *Telmatospirillum* |  |  |  |  | **1** | **2** | **2** |
|  | *Azospirillum* |  | **1** |  |  |  |  | **1** |
| **Sphingomonadales** | *Sphingomonas* |  |  |  |  |  |  | **2** |
| **Burkholderiales** | *Burkholderia* |  | **1** |  |  |  |  | **1** |
|  | *Ideonella* |  |  |  |  |  |  | **1** |
|  | *Pelomonas* |  |  |  |  |  |  | **1** |

*no subfamilial resolution
